# The antioxidant activity of natural diterpenes: theoretical insights†

**DOI:** 10.1039/d0ra02681f

**Published:** 2020-04-16

**Authors:** Quan V. Vo, Nguyen Minh Tam, Le Trung Hieu, Mai Van Bay, Nguyen Minh Thong, Trinh Le Huyen, Nguyen Thi Hoa, Adam Mechler

**Affiliations:** Institute of Research and Development, Duy Tan University Danang 550000 Vietnam vovanquan2@duytan.edu.vn; Faculty of Chemical Technology - Environment, The University of Danang-University of Technology and Education Danang 550000 Vietnam vvquan@ute.udn.vn; Computational Chemistry Research Group, Ton Duc Thang University Ho Chi Minh City Vietnam nguyenminhtam@tdtu.edu.vn; Faculty of Applied Sciences, Ton Duc Thang University Ho Chi Minh City Vietnam; University of Sciences-Hue University Hue 530000 Vietnam; Department of Chemistry, The University of Danang-, University of Science and Education Danang 550000 Vietnam; The University of Danang Campus in Kon Tum, 704 Phan Dinh Phung Kon Tum 580000 Vietnam; Department of Applied Chemistry, National Chiao Tung University Hsinchu 30010 Taiwan; Department of Chemical Engineering, The University of Danang-University of Science and Technology Danang 550000 Vietnam; Academic Affairs, The University of Danang-University of Technology and Education Danang 550000 Vietnam; Department of Chemistry and Physics, La Trobe University Victoria 3086 Australia

## Abstract

Diterpenes that were isolated from *Crossopetalum gaumeri* (Loes.) Lundell (Celastraceae) plants are reported to exhibit a range of biological activities, in particular as radical scavengers. Thus further insight into the antioxidant activity of diterpenes in physiological environments is much needed but not studied yet. In this study, the antioxidant activity of nine natural diterpenes was evaluated using kinetic and thermodynamic calculations. It was found that the sequential proton loss electron transfer (SPLET) mechanism is favored in polar environments, whereas formal hydrogen transfer (FHT) is the main pathway for the radical scavenging of these diterpenes in the gas phase as well as in lipid media. The rate constants for the HOO˙ radical scavenging of these compounds in the gas phase, polar and nonpolar solvents are in the range of 2.29 × 10^−2^ to 4.58 × 10^7^, 9.74 × 10^−3^ to 1.67 × 10^8^ and 3.54 × 10^−5^ to 1.31 × 10^5^ M^−1^ s^−1^, respectively. 7-Deoxynimbidiol (6), exhibits the highest HOO˙ radical scavenging with *k*_overall_ = 1.69 × 10^8^ M^−1^ s^−1^ and 9.10 × 10^4^ M^−1^ s^−1^ in water and pentyl ethanoate solvents, respectively, that is about 1300 times higher than that of Trolox in polar environments. It is thus a promising natural antioxidant in physiological environments.

## Abstract 2

Diterpenes that were isolated from *Crossopetalum gaumeri* (Loes.) Lundell (Celastraceae) plants are reported to exhibit a range of biological activities, in particular as radical scavengers.

## Graphical abstract

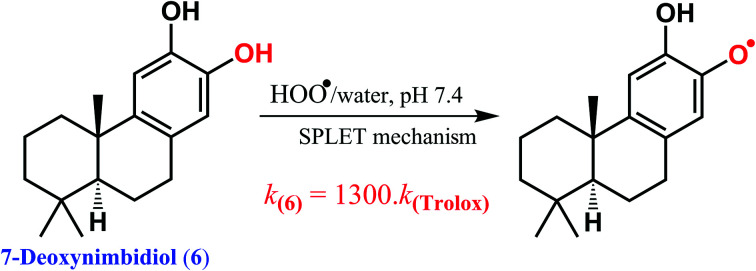

## 1. Introduction

**Fig. 1:**
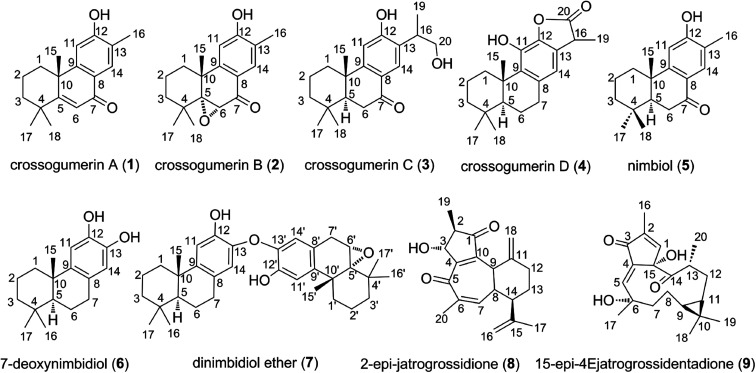
Structures of the nine diterpenes studied here for their antioxidant properties.

Diterpenes are natural products based on a podocarpane skeleton. These compounds are isolated from *Crossopetalum gaumeri* (Loes.) Lundell (Celastraceae) plants.^1^ Diterpenes and derivatives have great therapeutic potential.^2,3^ They exhibit a broad spectrum of biological activities including antimicrobial, antiviral, anti-inflammatory, and antitumor effects.^4^ Classes of diterpenes possessing high bio-activity include crossogumerins A–D (1–4), nimbiol (5), 7-deoxynimbidiol (6), dinimbidiol ether (7), 2-*epi*-jatrogrossidione (8), and 15-*epi*-4Ejatrogrossidentadione (9) (Fig. 1). Compounds 1–5 exhibit cytotoxicity against both HeLa (carcinoma of the cervix) and Hep-2 (lung carcinoma) human tumor cells lines, whereas compounds 2 and 5 exhibit strong activity against HeLa cells with low IC_50_ values (IC_50_ = 3.1 and 8.1 μM, respectively).^5^ Compound 6 showed good analgesic and anti-inflammatory activities, inhibiting the pain induced by PGE2 and reducing edema.^6^ Remarkably, the compounds 2-*epi*-jatrogrossidione (8) and 15-*epi*-4Ejatrogrossidentadione (9) exhibit potential antimicrobial activity against *Bacillus subtilis*.^1^

Experimental studies of the antioxidant properties of diterpenes revealed good activity,^1,6,7^ warranting a theoretical investigation of the free radical scavenging mechanism of these compounds. The antioxidant activity of some diterpenes including ferruginol, hinokiol and sugiol was evaluated theoretically by using the Density Functional Theory (DFT) method. It was showed that ferruginol and hinokiol could exhibit higher antioxidant activity than butylated hydroxytoluene (BHT).^8^ However the activity has not been fully explored thus far. Previous works demonstrated that quantum chemistry calculations offer an effective and elegant way to study the mechanism and kinetics of radical reactions, evaluating the antioxidant capacity of organic compounds at the molecular level in both gas phase and physiological environments.^8–16^ Thus a thermodynamic and kinetic study in the radical scavenging activity of the natural diterpenes is crucial to glean a better understanding of their radical scavenging activity.

This study is thus aimed to (1) investigate the thermodynamics of the antioxidant activity of diterpenes through three mechanisms: formal hydrogen transfer (FHT), sequential electron transfer proton transfer (SETPT), or sequential proton loss electron transfer (SPLET); (2) kinetically evaluate the HOO˙ radical scavenging capacity of diterpenes in gas phase and physiological environments; and (3) analyze the effects of solvent environments and molecular structures on the antioxidant activity and oxidation resistance of diterpene derivatives.

## 2. Computational methods

In this study, the thermochemical properties: bond dissociation energies (BDEs), ionization energies (IEs), and proton affinities (PAs) of all compounds were determined in the gas phase following the well-established (RO)B3LYP/6-311++G(2df,2p)//B3LYP/6-311G(d,p) calculating model.^12,17–22^ The kinetic study in the gas phase and physiological environments, including water for polar environment and pentyl ethanoate for non-polar environment with the solvation model density (SMD), was performed according to the quantum mechanics based test for overall free radical scavenging activity (QM-ORSA) protocol^10,23,24^ using the M06-2X/6-31+G(d,p) and M06-2X/6-311++G(d,p)^9,25–27^ levels and the Eyringpy code.^28,29^ The use of different functionals for thermochemistry and kinetics to enhance accuracy of the calculations was established and justified before.^11,13^ Rate constant (*k*) was computed following the transition state theory and 1 M standard state at 298.15 K according to the eqn (1) (details method in the Table S5, ESI†):^24,30–34^1
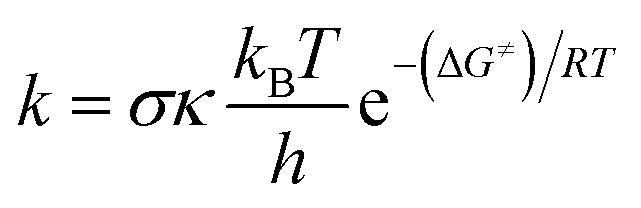
where *σ* the reaction symmetry number,^35,36^*κ* tunneling corrections which were calculated using Eckart barrier,^37^*k*_B_ the Boltzmann constant, *h* the Planck constant, Δ*G*^≠^ Gibbs free energy of activation.

The overall rate constant (*k*_overall_), and branching ratios (*Γ*) were computed following the QM-ORSA model.^10^ All of the calculations were performed with the Gaussian 16 suite of programs.^38^

## 3. Results and discussions

### 3.1. Thermodynamic study

According to previous studies, the antioxidant activity may follow either of three main mechanisms including FHT, SETPT or SPLET. Thermodynamically each of these are characterized by corresponding thermochemical parameters: BDEs, PAs, and IEs, respectively.^11,13,19,39^ While the actual values of these parameters may vary in solvent environments, the overall characteristics of the compounds established in the gas phase are normally transferable to different environments. Thus, in the initial step, the thermochemical characteristics of possible X–H (X = C, O) bonds were screened in the gas phase by using DFT calculation at the B3LYP/3-21G level (Table S1, ESI†). The lowest PA and BDE values were then calculated at higher level ROB3LYP/6-311++G(2df,2p)//B3LYP/6-311G(d,p)^11,19^ and these results are presented in Table 1.

**Table 1:** The calculated BDEs, Pas, and IEs (kcal mol^−1^) of the studied compounds in the gas phase

Comp.	Positions	BDE	PA	IE
1	O12–H	85.3	307.2	156.0
2	O12–H	85.4	305.1	156.3
3	O12–H	87.6	293.6	143.0
4	C16–H	74.6		146.7
	O11–H	84.0	306.0	146.7
5	O12–H	85.6	304.5	155.6
6	O12–H	74.7	311.0	140.9
	O13–H	75.2	310.7	
7	O12–H	83.0	273.4	111.2
8	C3–H	74.8		161.2
	C9–H		351.6	
9	C13–H	88.7		162.0
	C16–H		356.2	

It was found that the BDE values were in the range of 74.6 to 88.7 kcal mol^−1^. The radical scavenging of compounds 1, 2, 3, 5, 6, and 7 following FHT mechanism was defined by the O12(13)–H bonds, while the lowest BDE values of the rest compounds were observed at C–H bonds. Due to the lowest BDE(O–H) values were detected at the O12(13)–H bonds of the compound 6 (BDE = 74.7 and 75.2 kcal mol^−1^, respectively), this diterpene compound can be considered a promising radical scavenger following the FHT mechanism.

The calculated PA values of the studied compounds were in the range of 273.4–356.2 kcal mol^−1^ and the calculated IEs for the diterpenes were 111.2–162.0 kcal mol^−1^. Compound 7 has the lowest PA and IE values at 273.4 kcal mol^−1^ and 111.2 kcal mol^−1^, respectively, whereas those for 9 are highest at 356.2 kcal mol^−1^ and 162.0 kcal mol^−1^, respectively. Hence, the SETPT and SPLET mechanisms may be feasible for compound 7 in the gas phase.

The investigation of the free energy change (Δ*G*^o^) of the first step for the HOO˙ scavenging of the diterpenes (Table S2, ESI†) in the gas phase indicated that the SETPT and SPLET mechanisms are not spontaneous (Δ*G*^o^ > 0); the FHT mechanism, however, is supported by the negative Δ*G*^o^. Thus the FHT mechanism appears to be the main radical scavenging pathway for the studied diterpenes in the gas phase.

### 3.2. Kinetic study

#### 3.2.1. The reaction of HOO˙ radical with diterpenes following the formal hydrogen transfer mechanism in the gas phase

**Fig. 2:**
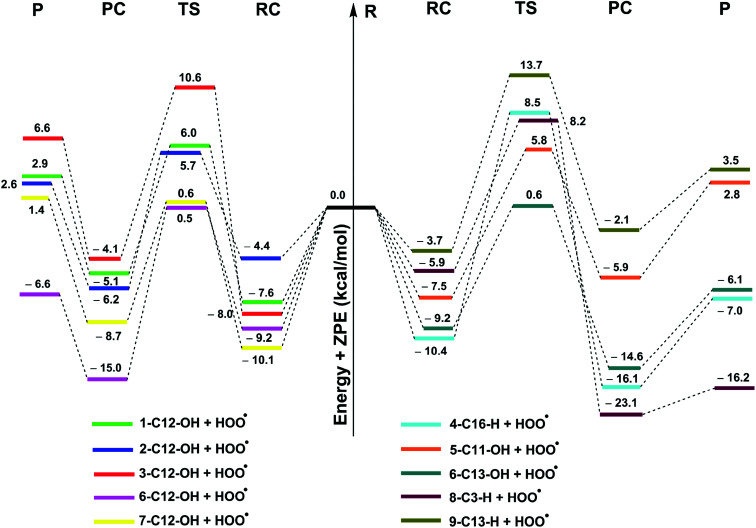
PES for the reactions of studied compounds with HOO˙ in the gas phase (reagent, RC: pre-complex; TS: transition state; PC: post-complex; P: products).

**Fig. 3:**
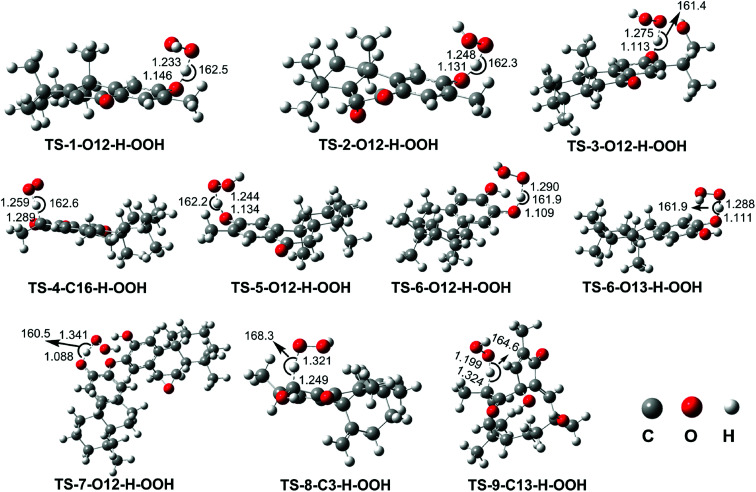
Optimized geometries TSs following the FHT mechanism between the studied compounds and HOO˙ radical in the gas phase.

As shown in the thermodynamic section, the HOO˙ radical scavenging of diterpenes in the gas phase was dominated by the FHT mechanism. Therefore, in this study the kinetic calculation focused on the H-abstraction of the studied compounds at the lowest BDE values. The potential energy surfaces (PES), the optimized structures of TSs and kinetic parameters, including Gibbs free energies of activation (Δ*G*^≠^, kcal mol^−1^), tunneling corrections (*κ*), and rate constants (*k*_Eck_, M^−1^ s^−1^), are presented in Fig. 2, 3 and Table 2.

**Table 2:** Calculated Δ*G*^≠^, *κ* and *k*_Eck_ for the HOO˙ scavenging of the diterpenes in the gas phase at the M06-2X/6-31+G(d,p) level

Reactions	Δ*G*^≠^	*κ*	*k*_Eck_
1–O12–H + HOO˙	14.2	210.7	5.18 × 10^4^
2–O12–H + HOO˙	14.3	144.4	2.77 × 10^4^
3–O12–H + HOO˙	19.4	16.9	6.63 × 10^−1^
4–C16–H + HOO˙	17.3	226.3	3.14 × 10^2^
5–O12–H + HOO˙	14.3	231.1	4.70 × 10^4^
6–O12–H + HOO˙	9.3	50.0	4.58 × 10^7^
6–O13–H + HOO˙	9.6	62.6	3.55 × 10^7^
7–O12–H + HOO˙	11.2	51.2	3.92 × 10^6^
8–C3–H + HOO˙	17.4	17.9	2.11 × 10^1^
9–C13–H + HOO˙	23.0	247.9	2.29 × 10^−2^

As shown in Fig. 2, the reaction proceeds *via* the pathway:R → RC → TS → PC → P

The RCs are energetically more stable than the reactants in the range of 2.1–23.1 kcal mol^−1^. After the formation of RC, the reactions can proceed to transition states (TS) by formal hydrogen transfer process with the reaction barriers in the range of 9.7–18.9 kcal mol^−1^ and then form the products after pass though the post-complexes. The H-abstraction of the 6–O12–H and 6–O13–H bonds is easier than those of the other compounds with the lowest reaction barriers (9.7 and 9.8 kcal mol^−1^, respectively). This suggests that the compound 6 is the best potential HOO˙ radical scavenger of all of the studied compounds.

As shown in Table 2, the reaction barriers for the HOO˙ scavenging of the diterpenes in the gas phase at 298.15 K are in the range of 9.3–23.0 kcal mol^−1^, while the rate constant values for these reactions are in the range of 2.29 × 10^−2^ to 4.58 × 10^7^ M^−1^ s^−1^ and the tunneling corrections (*κ*) for the H-abstraction of the X–H bonds are 16.9–247.9. The HOO˙ scavenging of 3, 8, and 9 are the lowest with *k*_Eck_ = 10^−2^ to 10^1^ M^−1^ s^−1^. However, the compounds 1, 2, 4, 5, and 7 exhibit a moderate HOO˙ radical scavenging activity with *k*_Eck_ = 10^2^ to 10^6^ M^−1^ s^−1^. It is important to note that the highest rate constants are observed at the reactions of 6 + HOO˙ with *k*_Eck_ = 4.58 × 10^7^ and 3.55 × 10^7^ M^−1^ s^−1^ for the H-abstraction of 6–O12–H and 6–O13–H, respectively. This result correlates well with the obtained BDE values in the thermodynamic investigation (BDE(O12–H) = 74.7 kcal mol^−1^, BDE(O13–H) = 75.2 kcal mol^−1^). The compound 6 is thus predicted as the most active antioxidant among all of the studied diterpenes in the gas phase.

#### 3.2.2. The HOO˙ scavenging of diterpenes in physiological environments

##### Acid–base equilibria

The investigation of radical scavenging in physiological environments provides the most accurate data of antioxidant activity.^10^ Therefore in this section the antioxidant activity of the diterpenes was evaluated against HOO˙ radical in physiological environments (water at pH = 7.4 for aqueous solution and pentyl ethanoate for lipid medium). As shown in the thermodynamic section, the lowest PA values for the compounds 8 and 9 were observed at the C–H bonds that have low p*K*_a_ (p*K*_a_ < 12) values. Consequently, these compounds were ignored in the acid–base equilibria calculations due to the neutral state at pH = 7.4. The p*K*_a_ values of seven diterpenes (1–7) were calculated using the model reaction (2) below, following the literature.^40^ The calculated p*K*_a_ values and the molar fractions (*f*) that were computed following the literature^10^ are presented in Table 3:

2 HA + Ref^−^ → A^−^ + HRef

**Table 3:** Calculated p*K*_a_ and *f* at pH = 7.4

Comp.	OH position	p*K*_a_	*f*_protonated_ (HA)	*f*_deprotonated_ (A^−^)
1	O12–H	8.30	0.888	0.112
2	O12–H	8.52	0.929	0.071
3	O12–H	8.20	0.863	0.137
4	O11–H	8.58	0.938	0.062
5	O12–H	8.55	0.934	0.066
6	O13–H	8.98	0.974	0.026
7	O12–H	7.77	0.701	0.299

The value of p*K*_a_ was defined by eqn (3):^40–42^

3 p*K*_a_ = Δ*G*_s_/*RT* ln(10) + p*K*_a_(HRef)

where the HRef is phenol with the experimental p*K*_a_(O–H) = 10.09.^43^

As shown in the Table 3, the calculated p*K*_a_ values are in the range of 7.77 to 8.98. The *f*_protonated_ (HA) and *f*_deprotonated_ (A^−^) are in the range of 0.701 to 0.974 and 0.026 to 0.299, respectively. Thus in the aqueous solution at pH = 7.4, the diterpenes 1–7 exist at both neutral and anion states, whereas 8 and 9 exist at neutral states and these states have been used for further study.

##### Kinetic study

From the thermodynamics section, the FHT mechanism defined the HOO˙ radical scavenging of the studied compounds in the gas phase. The FHT mechanism is thus a favorable pathway for the HOO˙ radical scavenging in the lipid solvent. However, in the aqueous solution, the diterpenes exist at both anion and neutral states, so the HOO˙ radical scavenging of diterpenes can follow both the FHT and SET mechanisms. Calculations of the Gibbs free energy change of the reactions of neutral diterpenes with HOO˙ radical following the SET mechanism yielded positive values in both water and lipid media (Table S3, ESI†) thus these reactions are not spontaneous. Hence, the HOO˙ radical scavenging in the physiological environments is calculated *via* the SET mechanism for the anion states and the FHT mechanism for the neutral states.^27^ The overall rate constants (*k*_overall_) were calculated according to the eqn (4) and (5) and the obtained results are shown in Table 4.

**Table 4:** The calculated Δ*G*^≠^ (in kcal mol^−1^), *k*_app_ (M^−1^ s^−1^) and *Γ* (%) of the reactions of the studied compounds with HOO˙ in water and pentyl ethanoate solvents at M06-2X/6-31+G(d,p) level

Comp.	Mechanism		Pentyl ethanoate			Water				
			Δ*G*^≠^	*k*_app_	*Γ*	Δ*G*^≠^	*k*_app_	*f*	*k*_f_ a	*Γ*
1	SET					9.9	3.60 × 10^5^	0.112	4.03 × 10^4^	100.0
	HAT	O12	19.4	1.27	100.0	18.9	1.89 × 10^1^	0.888	1.68 × 10^1^	0.0
	*k*_overall_			**1.27**					**4.03 × 10** ^**4**^	
2	SET					10.5	1.30 × 10^5^	0.071	9.23 × 10^3^	99.7
	HAT	O12	17.9	1.82 × 10^1^	100.0	18.7	2.64 × 10^1^	0.929	2.45 × 10^1^	0.3
	*k*_overall_			****1.82** × 10** ^**1**^					**9.25 × 10** ^**3**^	
3	SET					12	1.10 × 10^4^	0.137	1.51 × 10^3^	99.7
	HAT	O12	22.8	6.40 × 10^−3^	100.0	19.8	5.33	0.863	4.60	0.3
	*k*_overall_			**6.40 × 10** ^**−3**^					**1.51 × 10** ^**3**^	
4	SET					5.3	7.90 × 10^8^	0.062	4.90 × 10^7^	100.0
	HAT	C16	20.5	1.10	100.0	18.8	4.70 × 10^1^	0.938	4.41 × 10^1^	0.0
	*k*_overall_			**1.10**					**4.90 × 10** ^**7**^	
5	SET					10	3.00 × 10^5^	0.066	1.98 × 10^4^	99.9
	HAT	O11	17.8	2.39 × 10^1^	100.0	19.1	1.37 × 10^1^	0.934	1.28 × 10^1^	0.1
	*k*_overall_			**2.39 × 10** ^**1**^					**1.98 × 10** ^**4**^	
6	SET					3.2	6.40 × 10^9^	0.026	1.66 × 10^8^	99.9
	HAT	O12	12.9	7.70 × 10^4^	58.8	13.6	1.20 × 10^5^	0.974	1.17 × 10^5^	0.1
		C13	13.3	5.40 × 10^4^	41.2	14.4	6.70 × 10^4^		6.53 × 10^4^	0.0
	*k*_overall_			****1.31** × 10** ^**5**^					**1.67 × 10** ^**8**^	
7	SET					11.2	3.92 × 10^4^	0.299	1.17 × 10^4^	100.0
	HAT	O12	14.3	1.48 × 10^4^	100.0	15.5	2.07 × 10^3^	0.701	1.45 × 10^3^	0.0
	*k*_overall_			**1.48 × 10** ^**4**^					**1.32 × 10** ^**4**^	
8	SET									
	HAT	C3	20.3	2.80 × 10^−1^	100.0	19.2	3.00	1	3.00	100.0
	*k*_overall_			**2.80 × 10** ^**−1**^					**3.00**	
9	SET									
	HAT	C13	26.2	3.54 × 10^−5^	100.0	23.6	9.74 × 10^−3^	1	9.74 × 10^−3^	100.0
	*k*_overall_			**3.54 **× 10**** ^**−5**^					**9.74 × 10** ^**−3**^	

a *k*_f_ = *fk*_app_.

In lipid media:

4 *k*_overall_ = ∑*k*^FHT^_app_ (X–H)

where the X–H bonds are the O–H and C–H bonds that have the lowest BDE values.

In the aqueous solution:

5 *k*_overall_ = *f*_A^−^_*k*^SET^_app_ (A^−^) + *f*_HA_*k*^FHT^_app_ (HA) = *k*^SET^_f_ (A^−^) + *k*^FHT^_f_ (HA)

As can be seen in Table 4, the overall rate constants for the HOO˙ radical scavenging of the diterpenes in the lipid medium are in the range of 3.54 × 10^−5^ to 1.31 × 10^5^ M^−1^ s^−1^, whereas those for the aqueous solution are much higher at *k*_overall_ = 9.74 × 10^−3^ to 1.67 × 10^8^ M^−1^ s^−1^. The *k*_overall_ values in the lipid solvent are defined by the H-abstraction of the X–H bonds (*Γ* ∼ 100%), while the SET mechanism is the main pathway for the HOO˙ radical scavenging in the aqueous solution (*Γ* = 99.7–100.0%). The compounds 8 and 9 exhibit low HOO˙ radical scavenging activity in both water and pentyl ethanoate solvents with *k*_overall_ < 10^1^ that can be ascribed to the absence of phenolic system in these compounds. The compounds 1, 2, 3, 5, and 7 exhibit moderate HOO˙ radical scavenging activity in the aqueous solution (*k*_overall_ ∼ 10^3^ to 10^4^). Interestingly, the HOO˙ scavenging activity of compound 4 in the polar solvent is about 10^7^ times higher than that in the nonpolar solvent. Thus 4 has the second largest overall rate constant in the aqueous solution. The highest overall rate constant was calculated for compound 6 with *k*_overall_ = 1.31 × 10^5^ M^−1^ s^−1^ and 1.67 × 10^8^ M^−1^ s^−1^ in non-polar and polar media, respectively.

To gain more accurate values, the M06-2X/6-311++G(d,p) level of theory was used, which is currently among best methods to compute accurate kinetic parameters.^9,27^ At this level of theory the most active compound 6 was analyzed and the obtained results are presented in Table 5. It was found that the HOO˙ radical scavenging of 6 in water (*k*_overall_ = 1.69 × 10^8^ M^−1^ s^−1^) is about 1857 times higher than that (*k*_overall_ = 9.40 × 10^4^ M^−1^ s^−1^) in pentyl ethanoate solvent. Moreover, in comparison with a typical natural antioxidant-Trolox (*k*_overall_ = 1.00 × 10^5^ and 1.30 × 10^5^ M^−1^ s^−1^ in pentyl ethanoate and water, respectively), compound 6 exhibits similar HOO˙ radical scavenging activity in the lipid medium. However, the HOO˙ radical scavenging of 6 is about 1300 times higher than that of Trolox, and 13 times higher than that of *trans*-resveratrol^13^ in the polar environment. Hence, 6 is a promising antioxidant in polar environments.

**Table 5:** The calculated Δ*G*^≠^, *κ*, *k*_app_ of the HOO˙ radical scavenging of the best antioxidant in water and pentyl ethanoate solvents at M06-2X/6-311++G(d,p) level

Comp.	Mechanism		Pentyl ethanoate					Water					
			Δ*G*^≠^	*κ*		*k*_app_	*Γ*	Δ*G*^≠^	*λ*	*k*_app_	*f*	*k*_f_ a	*Γ*
6	SET						0.0	3.1	16.9	6.50 × 10^9^	0.026	1.69 × 10^8^	100.0
	HAT	O12	13.4		55.4	5.10 × 10^4^	56.0						0.0
		C13	13.8		83.8	4.00 × 10^4^	44.0						0.0
	*k*_overall_					**9.10 × 10** ^**4**^						**1.69 × 10** ^**8**^	
Trolox			12.6		28.8	1.00 × 10^5^		11.7				1.30 × 10^5^	

a *k*_f_ = *fk*_app_.

## 4. Conclusions

This study was carried out to evaluate the antioxidant activity of nine natural diterpenes by kinetic and thermodynamic calculations. It was found that the FHT mechanism is the main pathway for the radical scavenging of these diterpenes in the gas phase and in lipid environment, whereas the SET mechanism is favored in polar environment. The kinetic calculations showed that the rate constant values for the HOO˙ radical scavenging of these compounds in the gas phase, polar and nonpolar solvents are in the range of 2.29 × 10^−2^ to 4.58 × 10^7^ M^−1^ s^−1^, 9.74 × 10^−3^ to 1.67 × 10^8^ M^−1^ s^−1^ and 3.54 × 10^−5^ to 1.31 × 10^5^ M^−1^ s^−1^, respectively. The compounds 8 and 9 exhibited low radical scavenging activity due to the absence of phenolic system, whereas the phenolic compound 6 is predicted to be the most potent HOO˙ radical scavenger in all of the studied compounds with *k*_overall_ = 1.69 × 10^8^ M^−1^ s^−1^ (in water) and 9.10 × 10^4^ M^−1^ s^−1^ (in pentyl ethanoate solvent). Thus, compound 6 becomes one of the most potent antioxidant in polar physiological environments due to its HOO˙ radical scavenging is about 1300 and 13 times higher than those of Trolox and *trans*-resveratrol, respectively.

## Conflicts of interest

There are no conflicts to declare.

## Supplementary Material

RA-010-D0RA02681F-s001
