# Supplementary material for: The antioxidant activity of natural diterpenes: theoretical insights
Source: RSC Adv. 2020 Apr 16;10(25):14937–43. doi: 10.1039/d0ra02681f (PMC9052119; doi:10.1039/d0ra02681f)
Supplement: RA-010-D0RA02681F-s001 [file RA-010-D0RA02681F-s001.pdf]

Electronic Supplementary Material (ESI) for RSC Advances.
This journal is © The Royal Society of Chemistry 2020

Supporting Information (SI)

The Antioxidant Activity of Natural Diterpenes: Theoretical Insights

Quan V. Vo,[1,2*] Nguyen Minh Tam,[3,4*] Le Trung Hieu,[5] Mai Van Bay,[6] Nguyen Minh Thong,[7] Trinh Le Huyen[8] Nguyen Thi Hoa[9] and Adam Mechler[10]

[1]Institute of Research and Development, Duy Tan University, Danang 550000, Vietnam
[2]Faculty of Chemical Technology - Environment, The University of Danang - University of Technology and Education, Danang 550000, Vietnam.
[3]Computational Chemistry Research Group, Ton Duc Thang University, Ho Chi Minh City, Vietnam.
[4]Faculty of Applied Sciences, Ton Duc Thang University, Ho Chi Minh City, Vietnam
[5]Hue University of Sciences – Hue University, Hue 530000, Vietnam
[6]Department of Chemistry, The University of Danang - University of Education, Danang 550000, Vietnam
[7]The University of Danang, Campus in Kon Tum, 704 Phan Dinh Phung, Kon Tum, Vietnam
[8]Department of Applied Chemistry, National Chiao Tung University, Hsinchu 30010, Taiwan
[9]Academic Affairs, The University of Danang - University of Technology and Education, Danang 550000, Vietnam.
[10]Department of Chemistry and Physics, La Trobe University, Victoria 3086, Australia
*Corresponding author: vovanquan2@duytan.edu.vn; vvquan@ute.udn.vn; nguyenminhtam@tdtu.edu.vn

Table of Contents

Table S1. BDE and PA values of the X-H (X = O, C) bonds of the studied compounds in the gas phase at the B3LYP/3-21g level of theory S2

Table S2. The Calculated Free Energy (ΔGº, in kcal/mol at 298.15 k) of the Reaction Between the Selected Compounds with HOO• Radical via the Formal Hydrogen Transfer (FHT), Sequential Proton (SA) and SET Processes in the Gas Phase Using the (RO)B3LYP/6-311++g(2df,2p)//B3LYP/6-311g(d,p) Method. S6

Table S3. The Calculated Free Energy (ΔGº, in kcal/mol at 298.15 k) of the Reaction Between the neutral Compounds with HOO• Radical via the SET Processes in the water and pentyl ethanoate solvents S6

Table S4: The cartesian coordinates and energies of TS of the reaction between selected compounds with HOO• at the M06-2x/6-311++G(d,p) calculating method following the FHT mechanism S7

Table S5. The method to calculate rate constant following the conventional transition state theory S40

Table S1. BDE and PA values of the X-H (X = O, C) bonds of the studied compounds in the gas phase at the B3LYP/3-21g level of theory

Compounds	BDE		PA	
	**B3LYP/3-21G**	**(RO)B3LYP/6-311++G(2df,2p)//B3LYP/6-311G(d,p)**	**B3LYP/3-21G**	**(RO)B3LYP/6-311++G(2df,2p)//B3LYP/6-311G(d,p)**
1				
1-C1-H	97.4		317.7	
1-C2-H	96.5		287.6	
1-C3-H	95.5		293.8	
1-C6-H	107.9		317.7	
1-C11-H	111.5		318.6	
1-C12-OH-H	**77.6**	85.3	**265.0**	307.2
1-C14-H	113.0		322.0	
1-C15-H	89.2		294.6	
1-C18-H	100.2		326.9	
1-C19-H	97.8		293.7	
1-C20-H	97.6		293.0	
2				
2-C1-H	97.1		318.4	
2-C2-H	96.4		322.8	
2-C3-H	97.3		321.2	
2-C6-H	103.8		309.3	
2-C11-H	111.8		316.6	
2-C12-OH-H	**77.7**	85.4	**262.2**	305.1
2-C14-H	112.6		318.2	
2-C15-H	89.1		293.1	
2-C18-H	100.0		326.9	
2-C19-H	98.0		322.1	
2-C20-H	97.7		288.9	
3				
3-C1-H	97.7		323	
3-C2-H	96.3		328.9	
3-C3-H	97.3		326.7	
3-C5-H	92.3		306.9	
3-C6-H	87.4		288.3	
3-C11-H	111.4		317.1	
3-C12-OH-H	**85.3**	87.6	**259.2**	293.6

3-C14-H	110.5		317.7	
3-C15-H	87.9		293.6	
3-C16-H	97.4		311.6	
3-C16-OH-H	93.7		269.2	
3-C17-H	100.9		312.4	
3-C18-H	100.1		329.7	
3-C19-H	98.0		328	
3-C20-H	99.0		290.2	
4				
4-C1-H	89.9		275.9	
4-C2-H	95.8		273.8	
4-C3-H	96.4		274.7	
4-C5-H	90.1		268.9	
4-C6-H	95.6		273.9	
4-C7-H	83.5		266.8	
4-C11-OH-H	**71.1**	84.4	**263.9**	306.0
4-C14-H	103.8		298.7	
4-C16-H	**67.5**	74.6	270.2	
4-C18-H	100.0		281.8	
4-C19-H	97.3		281	
4-C20-H	93.4		281.2	
5			307.8	
5-C1-H	97.8		324.3	
5-C2-H	96.4		329.9	
5-C3-H	97.4		327.6	
5-C5-H	92.4		313.7	
5-C6-H	87.0		290.1	
5-C11-H	111.8		319.9	
5-C12-OH-H	**77.3**	85.6	**264.9**	304.5
5-C14-H	112.7		322.1	
5-C15-H	89.3		295.3	
5-C18-H	100.2		330.6	
5-C19-H	98.1		329	
5-C20-H	97.8		294.2	
6				
6-C1-H	97.5		330.5	
6-C2-H	95.2		335.4	
6-C3-H	97.0		332.3	
6-C5-H	91.1		321.2	
6-C6-H	94.7		329.5	

6-C7-H	81.9		294.1	
6-C11-H	111.4		324.8	
6-C12-OH-H	**68.1**	74.7	**272.4**	311.0
6-13-OH-H	**68.4**	75.2	**271.2**	310.7
6-C14-H	110.7		318.7	
6-C18-H	100.0		334.9	
6-C19-H	98.0		334.2	
6-C20-H	97.2		310.6	
7				
7-C1-H	94.4		327.4	
7-C1'-H	92.4		321	
7-C2-H	92.7		332.9	
7-C2'-H	93.7		328.1	
7-C3'-H	93.9		325.2	
7-C3-H	93.9		330.7	
7-C5'-H	90.2		274.7	
7-C5-H	91.2		318.7	
7-C6'-H	100.9		315.9	
7-C6-H	92.1		327.3	
7-C7'-H	103.7		307.5	
7-C7-H	84.3		290.9	
7-C11'-H	118.4		313.3	
7-C11-H	110.4		318.5	
7-C12'-OH-H	81.6		259.2	
7-C12-OH-H	**79.4**	83.0	**254.1**	273.5
7-C14'-H	99.0		309	
7-C14-H	98.2		265.3	
7-C18'-H	89.1		329.9	
7-C18-H	98.5		334	
7-C19'-H	80.9		326	
7-C19-H	80.6		332.6	
7-C20'-H	90.1		291.7	
7-C20-H	90.9		299.4	
8				
8-C2-H	85.6		277.7	
8-C3-H	**63.0**	74.7	267.5	
8-C3-OH-H	98.8		291.5	
8-C7-H	101.0		303.2	
8-C8-H	73.9		264.8	
8-C9-H	74.3		**256.9**	351.6

8-C12-H	85.1		297	
8-C13-H	97.1		314.1	
8-C14-H	81.2		287.5	
8-C16-H	107.3		266.9	
8-C17-H	86.8		302.3	
8-C18-H	110.0		275.2	
8-C19-H	100.7		133.3	
8-C20-H	88.0		296.1	
9				
9-C1-H	112.8		294.9	
9-C5-H	103.6		298.9	
9-C6-OH-H	103.0		293.2	
9-C7-H	96.4		275.6	
9-C8-H	92.3		307	
9-C9-H	97.2		261.7	
9-C11-H	99.3		275.3	
9-C12-H	92.4		289.4	
9-C13-H	**86.8**	88.7	261.5	
9-C15-OH-H	92.6		276.8	
9-C16-H	99.6		**256.7**	356.2
9-C17-H	89.2		276.5	
9-C18-H	89.4		322.9	
9-C19-H	98.3		326.5	
9-C20-H	112.8		286.6	

Table S2. The Calculated Free Energy (ΔG°, in kcal/mol at 298.15 k) of the Reaction Between the Selected Compounds with HOO• Radical via the Formal Hydrogen Transfer (FHT), Sequential Proton (SA) and SET Processes in the Gas Phase Using the (RO)B3LYP/6-311++g(2df,2p)//B3LYP/6-311g(d,p) Method.

Compounds	ΔG		
	FHT	PA	SET
1	-0.1	158.2	157.2
2	-0.1	156.1	158.1
3	2.1	145.2	150.8
4	-11.1	157.4	151.9
5	0.3	155.7	158.5
6	-9.9	162.5	143.9
7	-3.4	127.2	138.0
8	-24.6	153.5	189.5
9	3.0	159.5	160.0

Table S3. The Calculated Free Energy (ΔG°, in kcal/mol at 298.15 k) of the Reaction Between the neutral Compounds with HOO• Radical via the SET Processes in the water and pentyl ethanoate solvents

Compounds	ΔG	
	water	pentyl ethanoate
1	48.3	70.8
2	49.2	71.6
3	44.7	66.6
4	41.4	64.5
5	48.8	71.5
6	33.1	55.8
7	33.7	55.5
8	57.0	92.1
9	46.8	70.7

Table S4: The cartesian coordinates and energies of TS of the reaction between selected compounds with HOO• at the M06-2x/6-311++G(d,p) calculating method following the FHT mechanism

**Name**	**1-O12-H-OOH (gas phase)**
Cartesian Coordinates	Frequency and Energy
C 2.12261800 0.42586600 0.10753400<br>C 1.04260900 -0.66241100 0.07659900<br>C -0.35178000 -0.10738400 -0.22146200<br>C -0.65922700 1.26027000 -0.10997100<br>C 0.41423200 2.23596400 0.20576500<br>C 1.79083500 1.72638400 0.20214400<br>H -1.26597600 -2.04567600 -0.59006000<br>C -1.39914800 -0.97149700 -0.51902900<br>C -1.96249400 1.74523500 -0.30396800<br>H 2.53964200 2.50702000 0.27129100<br>C -3.00584000 0.89776400 -0.62341600<br>C -2.71065500 -0.49614700 -0.73923600<br>H -2.12057300 2.81520200 -0.19888200<br>O -3.66784200 -1.34107700 -1.05087500<br>H -4.28780800 -1.55287900 -0.11063500<br>C -4.41282400 1.36561400 -0.84858400<br>H -4.73496300 1.12860500 -1.86718000<br>H -5.11373900 0.84898400 -0.18191100<br>H -4.49566600 2.44207800 -0.68911600<br>O 0.16865300 3.41505900 0.41519900<br>C 1.40239600 -1.73199800 -0.98521500<br>H 1.24998600 -1.29587700 -1.98127100<br>H 0.71028900 -2.57445400 -0.89655300<br>C 2.83718100 -2.23614000 -0.87453800<br>H 2.98498600 -2.77035100 0.07157600<br>H 3.02387200 -2.96646100 -1.66864300<br>C 3.81725200 -1.07762600 -0.99733400<br>H 3.70616500 -0.62548700 -1.99299700<br>H 4.85242400 -1.43323700 -0.92420000<br>C 3.60702800 0.02233600 0.06438200<br>C 4.50362700 1.20839000 -0.32530800<br>H 4.56288800 1.96004700 0.46733000<br>H 5.51901400 0.83843500 -0.49903100<br>H 4.15530000 1.69339200 -1.24196800<br>C 4.10084400 -0.47039000 1.44356200<br>H 5.19521600 -0.51164500 1.43203000<br>H 3.79440400 0.22008800 2.23548200<br>H 3.74352400 -1.46783200 1.70117400<br>C 0.92873700 -1.32471300 1.47755000<br>H 0.74108700 -0.56600600 2.24302000<br>H 0.09051900 -2.02723500 1.47979200<br>H 1.82867800 -1.87428200 1.75004500<br>O -3.67135600 -0.99397100 1.73282700<br>H -3.92296700 -0.06797500 1.89585200<br>O -4.72303600 -1.52145600 1.04260000	Zero-point correction= 0.375059 (Hartree/Particle)<br>Thermal correction to Energy= 0.396544<br>Thermal correction to Enthalpy= 0.397488<br>Thermal correction to Gibbs Free Energy= 0.324463<br>Sum of electronic and zero-point Energies= -999.767032<br>Sum of electronic and thermal Energies= -999.745547<br>Sum of electronic and thermal Enthalpies= -999.744603<br>Sum of electronic and thermal Free Energies= -999.817628
**Name**	**1-O12-H-OOH (water)**
Cartesian Coordinates	Frequency and Energy
C 2.13772300 0.41722900 0.11245500<br>C 1.06442100 -0.67123700 0.09947700<br>C -0.33142900 -0.12399000 -0.18972800	Zero-point correction= 0.374533 (Hartree/Particle)<br>Thermal correction to Energy= 0.395741<br>Thermal correction to Enthalpy= 0.396685

C -0.64144300 1.24888700 -0.10949300<br>C 0.43407800 2.21678200 0.19999700<br>C 1.80286900 1.72221800 0.19861900<br>H -1.23261500 -2.07100200 -0.50622700<br>C -1.37716600 -0.99639300 -0.46456400<br>C -1.94119700 1.72967000 -0.33115400<br>H 2.55544900 2.50024100 0.26301600<br>C -2.98154600 0.87572500 -0.64703000<br>C -2.68207500 -0.52002100 -0.71249300<br>H -2.12169900 2.79821500 -0.26228300<br>O -3.63832500 -1.37941000 -1.03399900<br>H -4.32472700 -1.51035000 -0.14248200<br>C -4.37360700 1.35077100 -0.92278800<br>H -4.65625600 1.12730000 -1.95706500<br>H -5.09900800 0.83920900 -0.27981100<br>H -4.45131100 2.42689600 -0.76007800<br>O 0.18409200 3.41019300 0.40804700<br>C 1.41806400 -1.75104700 -0.95552600<br>H 1.26184300 -1.32375500 -1.95465300<br>H 0.72634100 -2.59088000 -0.84725100<br>C 2.85432400 -2.24825300 -0.84474900<br>H 3.00764300 -2.76634200 0.10908100<br>H 3.03712700 -2.98566800 -1.63304100<br>C 3.82828500 -1.08828700 -0.98938800<br>H 3.70816300 -0.64813900 -1.98920600<br>H 4.86575900 -1.43484900 -0.91248600<br>C 3.62165500 0.02376800 0.06060700<br>C 4.50670300 1.21035100 -0.34908700<br>H 4.56675800 1.97216500 0.43413800<br>H 5.52138000 0.83883200 -0.52336900<br>H 4.15140600 1.68133700 -1.27095600<br>C 4.12642000 -0.45038300 1.44216100<br>H 5.22129900 -0.46631100 1.42104500<br>H 3.81056800 0.23874800 2.23222400<br>H 3.79147400 -1.45481900 1.70348700<br>C 0.96988000 -1.32000500 1.50830400<br>H 0.76962500 -0.55768100 2.26749300<br>H 0.14834500 -2.04200400 1.51211900<br>H 1.88219700 -1.85005900 1.77743700<br>O -3.90993800 -0.82310700 1.73325000<br>H -4.26539100 0.08568700 1.77974800<br>O -4.82209500 -1.52786000 1.00587800	Thermal correction to Gibbs Free Energy= 0.324666<br>Sum of electronic and zero-point Energies= -999.790578<br>Sum of electronic and thermal Energies= -999.769371<br>Sum of electronic and thermal Enthalpies= -999.768427<br>Sum of electronic and thermal Free Energies= -999.840446
**Name**	**1-O12-H-OOH (pentyl ethanoate)**
Cartesian Coordinates	Frequency and Energy
C 2.12261800 0.42586600 0.10753400<br>C 1.04260900 -0.66241100 0.07659900<br>C -0.35178000 -0.10738400 -0.22146200<br>C -0.65922700 1.26027000 -0.10997100<br>C 0.41423200 2.23596400 0.20576500<br>C 1.79083500 1.72638400 0.20214400<br>H -1.26597600 -2.04567600 -0.59006000<br>C -1.39914800 -0.97149700 -0.51902900<br>C -1.96249400 1.74523500 -0.30396800	Zero-point correction= 0.375059 (Hartree/Particle)<br>Thermal correction to Energy= 0.396544<br>Thermal correction to Enthalpy= 0.397488<br>Thermal correction to Gibbs Free Energy= 0.324463<br>Sum of electronic and zero-point Energies= -999.767032<br>Sum of electronic and thermal Energies= -999.745547<br>Sum of electronic and thermal Enthalpies= -999.744603<br>Sum of electronic and thermal Free Energies= -999.817628

H 2.53964200 2.50702000 0.27129100<br>C -3.00584000 0.89776400 -0.62341600<br>C -2.71065500 -0.49614700 -0.73923600<br>H -2.12057300 2.81520200 -0.19888200<br>O -3.66784200 -1.34107700 -1.05087500<br>H -4.28780800 -1.55287900 -0.11063500<br>C -4.41282400 1.36561400 -0.84858400<br>H -4.73496300 1.12860500 -1.86718000<br>H -5.11373900 0.84898400 -0.18191100<br>H -4.49566600 2.44207800 -0.68911600<br>O 0.16865300 3.41505900 0.41519900<br>C 1.40239600 -1.73199800 -0.98521500<br>H 1.24998600 -1.29587700 -1.98127100<br>H 0.71028900 -2.57445400 -0.89655300<br>C 2.83718100 -2.23614000 -0.87453800<br>H 2.98498600 -2.77035100 0.07157600<br>H 3.02387200 -2.96646100 -1.66864300<br>C 3.81725200 -1.07762600 -0.99733400<br>H 3.70616500 -0.62548700 -1.99299700<br>H 4.85242400 -1.43323700 -0.92420000<br>C 3.60702800 0.02233600 0.06438200<br>C 4.50362700 1.20839000 -0.32530800<br>H 4.56288800 1.96004700 0.46733000<br>H 5.51901400 0.83843500 -0.49903100<br>H 4.15530000 1.69339200 -1.24196800<br>C 4.10084400 -0.47039000 1.44356200<br>H 5.19521600 -0.51164500 1.43203000<br>H 3.79440400 0.22008800 2.23548200<br>H 3.74352400 -1.46783200 1.70117400<br>C 0.92873700 -1.32471300 1.47755000<br>H 0.74108700 -0.56600600 2.24302000<br>H 0.09051900 -2.02723500 1.47979200<br>H 1.82867800 -1.87428200 1.75004500<br>O -3.67135600 -0.99397100 1.73282700<br>H -3.92296700 -0.06797500 1.89585200<br>O -4.72303600 -1.52145600 1.04260000	
**Name**	**2-O12-H-OOH (gas phase)**
Cartesian Coordinates	Frequency and Energy
C 2.08211500 0.31067400 -0.12537600<br>C 0.98083000 -0.70553700 0.25277600<br>C -0.39804400 -0.16249300 -0.13885400<br>C -0.70657700 1.20805700 0.03637900<br>C 0.37541900 2.16427800 0.37924800<br>C 1.74248000 1.75307000 -0.06490100<br>H -1.29095900 -2.05977000 -0.68899100<br>C -1.42964500 -0.99647800 -0.52854500<br>C -1.99725500 1.70587700 -0.15608400<br>C -3.03859900 0.88204700 -0.55707200<br>C -2.73696700 -0.49802500 -0.76065700<br>H -2.16239600 2.76881200 -0.00103500<br>O -3.66387700 -1.32593400 -1.18678700<br>H -4.47125400 -1.43735800 -0.40255300<br>C -4.42977600 1.38135200 -0.79603400<br>H -4.79184100 1.05103000 -1.77393200	Zero-point correction= 0.379806 (Hartree/Particle)<br>Thermal correction to Energy= 0.401884<br>Thermal correction to Enthalpy= 0.402828<br>Thermal correction to Gibbs Free Energy= 0.329396<br>Sum of electronic and zero-point Energies= -1074.943661<br>Sum of electronic and thermal Energies= -1074.921584<br>Sum of electronic and thermal Enthalpies= -1074.920639<br>Sum of electronic and thermal Free Energies= -1074.994072

H -5.11695800 0.97870600 -0.04417100<br>H -4.46258300 2.47118200 -0.74915400<br>O 0.18510800 3.22200400 0.94748800<br>C 1.27869000 -2.03514600 -0.47095100<br>H 1.09148100 -1.89544400 -1.54391200<br>H 0.58455600 -2.80500500 -0.11738000<br>C 2.71508100 -2.51889700 -0.27205800<br>H 2.88922600 -2.77886800 0.77898000<br>H 2.86104800 -3.44367900 -0.83954700<br>C 3.71997200 -1.46832500 -0.73612900<br>H 3.58961200 -1.30455000 -1.81490900<br>H 4.74670100 -1.82582200 -0.58997300<br>C 3.56509200 -0.11103100 -0.02140300<br>C 4.47188900 0.90442300 -0.73458600<br>H 4.54931600 1.84532500 -0.18088300<br>H 5.48088500 0.48492500 -0.79632500<br>H 4.12130200 1.11851900 -1.74580200<br>H 2.51390600 2.50544300 0.07605200<br>O 1.77402000 1.04194300 -1.30212100<br>C 0.91426000 -0.92646400 1.78199500<br>H 1.77085400 -1.48089100 2.16371900<br>H 0.85669600 0.02807500 2.31598200<br>H 0.01409500 -1.50084500 2.01977700<br>C 4.03918600 -0.19629000 1.44021500<br>H 3.72117600 0.68481500 2.00832800<br>H 3.68622000 -1.08476800 1.96438500<br>H 5.13344800 -0.22738000 1.45656500<br>O -4.29261500 -0.75351100 1.50995300<br>H -3.77173000 -1.38578100 2.03296600<br>O -5.05660000 -1.54115400 0.69447200	
**Name**	**2-O12-H-OOH (water)**
Cartesian Coordinates	Frequency and Energy
C 2.04748300 0.31747700 -0.13483500<br>C 0.93501600 -0.69611900 0.20965800<br>C -0.43135000 -0.12791900 -0.18008000<br>C -0.73019000 1.24070300 0.03419900<br>C 0.36285300 2.16103100 0.41873700<br>C 1.72463200 1.76305300 -0.03435900<br>H -1.33189700 -2.00349500 -0.77750300<br>C -1.46734000 -0.94232300 -0.59957900<br>C -2.01743100 1.75789600 -0.13897100<br>C -3.06536400 0.94878100 -0.55331200<br>C -2.76812400 -0.42287700 -0.80516200<br>H -2.18816600 2.81561800 0.04256800<br>O -3.72536100 -1.23070100 -1.23821700<br>H -4.36013000 -1.52802300 -0.35160200<br>C -4.46057800 1.45273800 -0.73869800<br>H -4.78515000 1.32206000 -1.77611300<br>H -5.15743600 0.88616400 -0.11085000<br>H -4.52611100 2.50991300 -0.47687400<br>O 0.18127900 3.19516000 1.04885300<br>C 1.22540300 -2.01444900 -0.53707100<br>H 1.05609300 -1.85539800 -1.61058800<br>H 0.51711300 -2.77913100 -0.20320600	Zero-point correction= 0.378701 (Hartree/Particle)<br>Thermal correction to Energy= 0.400640<br>Thermal correction to Enthalpy= 0.401584<br>Thermal correction to Gibbs Free Energy= 0.328428<br>Sum of electronic and zero-point Energies= -1074.968715<br>Sum of electronic and thermal Energies= -1074.946776<br>Sum of electronic and thermal Enthalpies= -1074.945832<br>Sum of electronic and thermal Free Energies= -1075.018988

C 2.65347900 -2.51466600 -0.32526800<br>H 2.80952900 -2.78978000 0.72423400<br>H 2.79710900 -3.42993400 -0.90828900<br>C 3.67425600 -1.46759000 -0.75922800<br>H 3.56153200 -1.28865100 -1.83786900<br>H 4.69577900 -1.83274100 -0.59906300<br>C 3.52410500 -0.11860300 -0.02810400<br>C 4.45195100 0.89593400 -0.71140900<br>H 4.49585600 1.84567500 -0.16929200<br>H 5.46401000 0.47945400 -0.71869700<br>H 4.15724400 1.09118300 -1.74491300<br>H 2.50505000 2.50018100 0.13082900<br>O 1.74928100 1.08951400 -1.29765300<br>C 0.84460400 -0.94948900 1.73306800<br>H 1.67670500 -1.54546500 2.10621900<br>H 0.81627900 -0.00674800 2.29062700<br>H -0.07829000 -1.49840500 1.94440800<br>C 3.97170100 -0.22673300 1.43941700<br>H 3.65918400 0.65521700 2.00966400<br>H 3.59481200 -1.11596600 1.94540400<br>H 5.06508500 -0.27661000 1.46836200<br>O -3.93865500 -0.94713200 1.55393300<br>H -3.20459100 -1.53561600 1.81908200<br>O -4.77782300 -1.72691200 0.81676400	
**Name**	**2-O12-H-OOH (pentyl ethanoate)**
Cartesian Coordinates	Frequency and Energy
C 2.07396000 0.31099300 -0.12972600<br>C 0.97003500 -0.70433100 0.24308900<br>C -0.40541800 -0.15291200 -0.14589000<br>C -0.70895100 1.21856700 0.03827900<br>C 0.37900200 2.16113900 0.39964500<br>C 1.74191300 1.75537800 -0.05365300<br>H -1.30032000 -2.04397000 -0.70572800<br>C -1.43979400 -0.98060100 -0.54405000<br>C -1.99694900 1.72457200 -0.15463100<br>C -3.04081600 0.90618900 -0.56161000<br>C -2.74328300 -0.47331900 -0.77287400<br>H -2.16599700 2.78665000 0.00208600<br>O -3.67965900 -1.29218200 -1.20660900<br>H -4.44438100 -1.45000500 -0.39867000<br>C -4.42858200 1.41365200 -0.79603900<br>H -4.75681400 1.18531900 -1.81515500<br>H -5.13834600 0.93278500 -0.11397200<br>H -4.47562800 2.49315300 -0.64109700<br>O 0.19193400 3.20632600 0.99725300<br>C 1.26357800 -2.03273400 -0.48507700<br>H 1.07993900 -1.89297500 -1.55887300<br>H 0.56604200 -2.79979900 -0.13286000<br>C 2.69767200 -2.52079500 -0.28306700<br>H 2.86789100 -2.78144300 0.76837600<br>H 2.84114400 -3.44414300 -0.85391600<br>C 3.70766500 -1.47357500 -0.74260100<br>H 3.58426700 -1.31143900 -1.82275300<br>H 4.73261600 -1.83312200 -0.58906500	Zero-point correction= 0.379168 (Hartree/Particle)<br>Thermal correction to Energy= 0.401204<br>Thermal correction to Enthalpy= 0.402148<br>Thermal correction to Gibbs Free Energy= 0.328934<br>Sum of electronic and zero-point Energies= -1074.971109<br>Sum of electronic and thermal Energies= -1074.949074<br>Sum of electronic and thermal Enthalpies= -1074.948129<br>Sum of electronic and thermal Free Energies= -1075.021343

<table>
<tr><td>C 3.55572500 -0.11544900 -0.02829000
C 4.46716000 0.89749800 -0.73698700
H 4.53529600 1.84366000 -0.19026800
H 5.47785000 0.47922800 -0.78292800
H 4.13361300 1.10333900 -1.75635100
H 2.51941300 2.50057300 0.09286800
O 1.76440900 1.05702000 -1.30025300
C 0.90085500 -0.93374500 1.77071500
H 1.75270700 -1.49889800 2.14771800
H 0.85067200 0.01663400 2.31392700
H -0.00329300 -1.50427200 2.00515000
C 4.02582500 -0.20294800 1.43389500
H 3.71259700 0.68010300 2.00246800
H 3.66774900 -1.09066100 1.95642200
H 5.12036300 -0.23965700 1.45094300
O -4.22991300 -0.81031400 1.52920700
H -3.63417600 -1.43540900 1.98298200
O -5.00186800 -1.59598900 0.72189300</td><td></td></tr>
<tr><td>Name</td><td>3-O12-H-OOH (gas phase)</td></tr>
<tr><td>Cartesian Coordinates</td><td>Frequency and Energy</td></tr>
<tr><td>C -2.62816700 0.52388400 0.14851300
C -1.74494200 -0.72188500 -0.17125100
C -0.29297500 -0.34170800 0.13891200
C 0.18267300 0.96924700 -0.09969600
C -0.73365400 2.04879700 -0.58080000
C -2.20330600 1.71577600 -0.71210300
H 0.35220300 -2.30125500 0.80393000
C 0.62681600 -1.27331900 0.59264300
C 1.52439100 1.31139200 0.11851000
C 2.45041300 0.39377800 0.58131000
C 1.98132000 -0.93038700 0.82698900
H 1.81319300 2.33787100 -0.09241600
O 2.79376800 -1.86027800 1.29168700
H 3.49986200 -2.14425500 0.47958400
C 3.88855800 0.76845500 0.84994300
H 4.42484000 -0.14235300 1.13867400
O -0.31225200 3.15745400 -0.84997600
C -2.19804900 -1.90244400 0.71043500
H -1.93074200 -1.68728500 1.75464300
H -1.65673800 -2.81074100 0.42272600
C -3.70163900 -2.16146700 0.63114400
H -3.98326200 -2.47386000 -0.38185600
H -3.95824200 -2.99846600 1.28902400
C -4.48543500 -0.91948700 1.04335100
H -4.24254400 -0.68369000 2.09009800
H -5.56483600 -1.11382900 1.00803400
C -4.17172100 0.31541500 0.17937200
C -4.82283600 1.53294000 0.85857800
H -4.79104800 2.42641800 0.22811600
H -5.87706700 1.31968500 1.06598400
H -4.33398200 1.76651200 1.81090900
H -2.39405100 1.52377100 -1.77539500
C -1.76081200 -1.15798200 -1.65378900
H -2.74068900 -1.51356400 -1.97443100</td><td>Zero-point correction= 0.461674 (Hartree/Particle)
Thermal correction to Energy= 0.486899
Thermal correction to Enthalpy= 0.487843
Thermal correction to Gibbs Free Energy= 0.407152
Sum of electronic and zero-point Energies= -1154.683234
Sum of electronic and thermal Energies= -1154.658008
Sum of electronic and thermal Enthalpies= -1154.657064
Sum of electronic and thermal Free Energies= -1154.737755</td></tr>
</table>

H -1.45255200 -0.35217200 -2.32547000<br>H -1.04805700 -1.97832100 -1.78792300<br>C -4.82143000 0.16794200 -1.20628500<br>H -4.55823500 0.99971600 -1.86715200<br>H -4.55773600 -0.76176000 -1.71283700<br>H -5.91107200 0.17665400 -1.09283800<br>H -2.36791100 0.77613900 1.19048500<br>H -2.75095600 2.62716600 -0.45973200<br>C 4.57504700 1.34132100 -0.39659000<br>H 4.11608500 2.29816700 -0.67126800<br>H 5.62743600 1.54328300 -0.14435900<br>O 4.46838300 0.51439800 -1.52951900<br>H 4.67059900 -0.40081400 -1.28843800<br>C 3.98837400 1.76469800 2.01367100<br>H 3.45470400 2.69164400 1.77907600<br>H 3.55475400 1.34390300 2.92425400<br>H 5.03466400 2.01440800 2.21460800<br>O 2.95639700 -1.99706100 -1.50318700<br>H 2.50485500 -2.83655100 -1.69638400<br>O 3.98755000 -2.35404100 -0.67985300	
**Name**	**3-O12-H-OOH (water)**
Cartesian Coordinates	Frequency and Energy
C -2.62704400 0.50190000 0.21834800<br>C -1.78047800 -0.71512100 -0.26239000<br>C -0.31754900 -0.40233600 0.06060900<br>C 0.18590900 0.92189600 -0.03719800<br>C -0.71751800 2.04950100 -0.40186700<br>C -2.19268200 1.77880300 -0.50247600<br>H 0.28352200 -2.43680600 0.47953700<br>C 0.58465000 -1.39846800 0.39214400<br>C 1.53481800 1.21162900 0.19754100<br>C 2.44690600 0.22332400 0.53794600<br>C 1.94454400 -1.10857200 0.65065300<br>H 1.85852100 2.24371100 0.10270900<br>O 2.74748200 -2.09690000 1.02040700<br>H 3.42359300 -2.36941000 0.16060700<br>C 3.89852500 0.50999100 0.83171200<br>H 4.47514800 -0.39178800 0.59375200<br>O -0.26599400 3.17022900 -0.62007600<br>C -2.24187300 -1.97983500 0.48825800<br>H -1.95499000 -1.88778200 1.54525200<br>H -1.72398100 -2.85757700 0.08705000<br>C -3.75197600 -2.19564100 0.40813500<br>H -4.05585400 -2.38375300 -0.62879200<br>H -4.01467100 -3.09541400 0.97472100<br>C -4.50117400 -0.99259700 0.97202600<br>H -4.23380500 -0.87907700 2.03305700<br>H -5.58543600 -1.15723700 0.93196700<br>C -4.17347900 0.32344800 0.24503500<br>C -4.78754500 1.47212700 1.06189100<br>H -4.74058600 2.42802600 0.53069300<br>H -5.84427500 1.25825800 1.25799900<br>H -4.28021100 1.58804500 2.02651400<br>H -2.42462800 1.73226000 -1.57459700	Zero-point correction= 0.459741 (Hartree/Particle)<br>Thermal correction to Energy= 0.485166<br>Thermal correction to Enthalpy= 0.486110<br>Thermal correction to Gibbs Free Energy= 0.405252<br>Sum of electronic and zero-point Energies= -1154.715024<br>Sum of electronic and thermal Energies= -1154.689599<br>Sum of electronic and thermal Enthalpies= -1154.688655<br>Sum of electronic and thermal Free Energies= -1154.769512

C -1.82828100 -0.97699700 -1.78408600<br>H -2.81841000 -1.29021100 -2.11709300<br>H -1.53320300 -0.09761400 -2.36432800<br>H -1.12589200 -1.78194700 -2.02503700<br>C -4.84283900 0.34086800 -1.13823300<br>H -4.56025700 1.22798200 -1.71450100<br>H -4.61625200 -0.54109400 -1.73989600<br>H -5.93025500 0.37178300 -1.00378100<br>H -2.34976900 0.62495500 1.27813200<br>H -2.70271100 2.66428600 -0.11319000<br>C 4.46631300 1.64989500 -0.01040900<br>H 3.99878200 2.60450700 0.25077200<br>H 5.53810600 1.73400600 0.20906000<br>O 4.26069700 1.46868300 -1.40801500<br>H 4.42869700 0.53949700 -1.62523500<br>C 4.08854200 0.82176300 2.32292600<br>H 3.52044000 1.71600400 2.59996600<br>H 3.74310800 -0.01165200 2.94053100<br>H 5.14585900 0.99974800 2.54192600<br>O 3.44550000 -1.42134500 -1.64412200<br>H 2.65901300 -1.75379100 -2.11924100<br>O 3.94903700 -2.49709000 -0.97593800	
**Name**	**3-O12-H-OOH (pentyl ethanoate)**
Cartesian Coordinates	Frequency and Energy
C -2.62702500 0.52706000 0.14735900<br>C -1.74087400 -0.71985900 -0.15627200<br>C -0.29063700 -0.33104400 0.14860400<br>C 0.18166100 0.98030200 -0.09543800<br>C -0.73909000 2.04861800 -0.59319200<br>C -2.20293600 1.71001200 -0.72560100<br>H 0.35883000 -2.28904700 0.80906800<br>C 0.63353700 -1.25988900 0.60308100<br>C 1.52344000 1.32745900 0.12373400<br>C 2.45239800 0.41325700 0.58729300<br>C 1.98644900 -0.91184500 0.83432500<br>H 1.81786900 2.35280000 -0.08505200<br>O 2.80549600 -1.83760900 1.30226200<br>H 3.48630200 -2.14717600 0.48329500<br>C 3.89175800 0.79335300 0.84548500<br>H 4.43148500 -0.10901500 1.15356500<br>O -0.31555700 3.15573100 -0.88115800<br>C -2.19071400 -1.88999400 0.74117000<br>H -1.92838500 -1.65895800 1.78339900<br>H -1.64623300 -2.80032300 0.46650300<br>C -3.69347300 -2.15321600 0.66019700<br>H -3.96969500 -2.47798900 -0.35047000<br>H -3.94909800 -2.98174300 1.32970500<br>C -4.48349200 -0.90872000 1.05188600<br>H -4.24657000 -0.65689000 2.09635100<br>H -5.56217600 -1.10719900 1.01135000<br>C -4.16989600 0.31450800 0.17156200<br>C -4.82768300 1.53982900 0.82838500<br>H -4.79918000 2.42310800 0.18246700<br>H -5.88177100 1.32468100 1.03750400	Zero-point correction= 0.460695 (Hartree/Particle)<br>Thermal correction to Energy= 0.485917<br>Thermal correction to Enthalpy= 0.486861<br>Thermal correction to Gibbs Free Energy= 0.406371<br>Sum of electronic and zero-point Energies= -1154.713265<br>Sum of electronic and thermal Energies= -1154.688044<br>Sum of electronic and thermal Enthalpies= -1154.687099<br>Sum of electronic and thermal Free Energies= -1154.767590

H -4.34164400 1.79291700 1.77783800	
H -2.38386900 1.50319300 -1.78823800	
C -1.75305600 -1.17362500 -1.63274300	
H -2.72868200 -1.54959600 -1.94396000	
H -1.46244800 -0.36891900 -2.31448400	
H -1.02808700 -1.98495300 -1.76122900	
C -4.81057000 0.14337900 -1.21489900	
H -4.53842900 0.95990400 -1.89184600	
H -4.54970800 -0.79883200 -1.70048400	
H -5.90143900 0.16112700 -1.10823400	
H -2.37389300 0.79250900 1.18726900	
H -2.75942400 2.62005900 -0.48648600	
C 4.57440400 1.33717800 -0.41358500	
H 4.11023600 2.28336500 -0.71479200	
H 5.62609500 1.54712000 -0.16883800	
O 4.47871500 0.47303500 -1.52794300	
H 4.61903500 -0.44145100 -1.24152700	
C 3.99795400 1.81875100 1.98188000	
H 3.47064000 2.74339100 1.72390600	
H 3.56264300 1.42379500 2.90394100	
H 5.04655800 2.06534900 2.17626400	
O 2.91923700 -2.06720700 -1.49949200	
H 2.43937800 -2.90719600 -1.62907900	
O 3.96320200 -2.39234000 -0.68080600	
**Name**	**4-C16-H-OOH (gas phase)**
Cartesian Coordinates	Frequency and Energy
C -2.81541900 0.63864300 0.21887100	Zero-point correction= 0.439857 (Hartree/Particle)
C -1.92550000 -0.52622200 -0.31418500	Thermal correction to Energy= 0.463952
C -0.45281200 -0.06272100 -0.30050700	Thermal correction to Enthalpy= 0.464896
C -0.08592800 1.30187100 -0.19876500	Thermal correction to Gibbs Free Energy= 0.387461
C -1.12558200 2.40335300 -0.12797400	Sum of electronic and zero-point Energies= -1153.510109
C -2.51368800 1.93300800 -0.53591700	Sum of electronic and thermal Energies= -1153.486015
C 0.58601200 -1.01099600 -0.46172800	Sum of electronic and thermal Enthalpies= -1153.485071
C 1.25404500 1.71046200 -0.17479900	Sum of electronic and thermal Free Energies= -1153.562506
H -3.24245800 2.71325200 -0.30142600	
C 2.26022200 0.75973500 -0.26959500	
C 1.89234300 -0.56993900 -0.41814900	
H 1.49055800 2.76765700 -0.08943300	
O 2.97646700 -1.41470100 -0.58513600	
C 3.72675900 0.79025100 -0.28500500	
C -2.13966700 -1.72405100 0.64680300	
H -1.73849800 -1.44903300 1.63298000	
H -1.57916100 -2.59316700 0.30468200	
C -3.61641300 -2.09866700 0.79558000	
H -4.00822700 -2.47319300 -0.15796500	
H -3.70105400 -2.93310700 1.50048000	
C -4.45774000 -0.92625000 1.28847800	
H -4.13649100 -0.67057400 2.30923400	
H -5.51633000 -1.21018200 1.35500300	
C -4.32758300 0.33616300 0.41648100	
C -4.96897900 1.49589100 1.20052400	
H -5.11724700 2.38598900 0.58202000	
H -5.95477800 1.18889400 1.56707600	
H -4.36018900 1.77620800 2.06792000	

C -5.12101200 0.18223000 -0.89172000<br>H -6.19313500 0.25299300 -0.67652600<br>H -4.87418400 0.97799000 -1.60342700<br>H -4.95411400 -0.77538600 -1.38647400<br>C -2.23133200 -0.95932100 -1.76672300<br>H -2.27218000 -0.10342300 -2.44593000<br>H -1.44511000 -1.63042300 -2.12052900<br>H -3.17510100 -1.49818400 -1.84603800<br>H -1.16997300 2.78251800 0.90235700<br>H -0.79225500 3.23898600 -0.75283300<br>H -2.55712700 1.77607700 -1.61969300<br>H -2.44812000 0.80074700 1.24804400<br>C 4.11213300 -0.63459700 -0.56610500<br>O 5.21066300 -1.10905600 -0.68202100<br>O 0.33450700 -2.33052200 -0.69191200<br>H 1.17519500 -2.80137500 -0.77681900<br>C 4.52864200 1.88609800 -0.94730700<br>H 5.59594900 1.67303500 -0.85789100<br>H 4.27024400 1.96094600 -2.00853700<br>H 4.32175400 2.84919700 -0.47441100<br>H 4.15672200 0.81402300 0.93022100<br>O 4.96799200 -0.64727300 2.16561200<br>H 5.73365100 -0.93183900 1.63380800<br>O 4.88795200 0.69676900 1.94777700	
**Name**	**4-C16-H-OOH (water)**
Cartesian Coordinates	Frequency and Energy
C 2.65080000 -0.66193000 0.02679300<br>C 1.78304800 0.59399600 -0.29899500<br>C 0.29796400 0.16685000 -0.33270200<br>C -0.09525600 -1.19153100 -0.44273500<br>C 0.91815500 -2.31131300 -0.56837000<br>C 2.30979000 -1.81144300 -0.91996400<br>C -0.72412600 1.15169200 -0.32026700<br>C -1.44300500 -1.57544600 -0.45802000<br>H 3.02228900 -2.63429400 -0.82201600<br>C -2.42843200 -0.60039500 -0.38808000<br>C -2.03780800 0.73105700 -0.32263600<br>H -1.70463500 -2.62725200 -0.53557400<br>O -3.12578300 1.60037300 -0.32238500<br>C -3.89148900 -0.61996800 -0.32391700<br>C 2.04443500 1.61694400 0.83695300<br>H 1.64786100 1.19903300 1.77354200<br>H 1.51189300 2.54787700 0.64713000<br>C 3.53236900 1.92630500 1.02044600<br>H 3.92245300 2.43572800 0.13097800<br>H 3.64497800 2.63434000 1.84921000<br>C 4.34713800 0.67033200 1.30470700<br>H 4.02911400 0.25820000 2.27395300<br>H 5.41376300 0.91387600 1.39754700<br>C 4.17203000 -0.42700300 0.24023500<br>C 4.79863800 -1.71194500 0.80927000<br>H 4.91118100 -2.49217500 0.05042700<br>H 5.79901700 -1.48855400 1.19764500<br>H 4.19807500 -2.11622700 1.63280400	Zero-point correction= 0.437748 (Hartree/Particle)<br>Thermal correction to Energy= 0.462118<br>Thermal correction to Enthalpy= 0.463062<br>Thermal correction to Gibbs Free Energy= 0.384826<br>Sum of electronic and zero-point Energies= -1153.530847<br>Sum of electronic and thermal Energies= -1153.506477<br>Sum of electronic and thermal Enthalpies= -1153.505533<br>Sum of electronic and thermal Free Energies= -1153.583769

C 4.94354500 -0.07594900 -1.04123800	
H 6.01883200 -0.18449800 -0.85692600	
H 4.67854900 -0.75339800 -1.86125400	
H 4.77404500 0.94765300 -1.37950300	
C 2.08521500 1.24184400 -1.67057900	
H 2.17323200 0.49279000 -2.46255300	
H 1.27608000 1.92239600 -1.94849500	
H 3.00532600 1.82691000 -1.65458500	
H 0.96665700 -2.84966700 0.38776500	
H 0.55176100 -3.02765300 -1.31086300	
H 2.34381400 -1.48492600 -1.96557300	
H 2.29622800 -0.97878900 1.02338500	
C -4.26385900 0.82786300 -0.42731400	
O -5.35030300 1.34127500 -0.53994100	
O -0.44187500 2.48841700 -0.33442800	
H -1.26832200 2.99420100 -0.37124400	
C -4.74979500 -1.63705500 -1.03254900	
H -5.80046900 -1.50162200 -0.76681000	
H -4.64445900 -1.52912100 -2.11688400	
H -4.44180000 -2.64627100 -0.75083700	
H -4.10403200 -0.78429200 0.91072200	
O -2.82769400 -0.70590300 2.57339700	
H -2.71466600 0.25678000 2.68600300	
O -4.12970900 -0.86772300 2.21032900	
**Name**	**4-C16-H-OOH (pentyl ethanoate)**
Cartesian Coordinates	Frequency and Energy
C -2.80889900 0.64010800 0.21722200	Zero-point correction= 0.438737 (Hartree/Particle)
C -1.91935800 -0.52837800 -0.31019100	Thermal correction to Energy= 0.462945
C -0.44427400 -0.06892100 -0.29416500	Thermal correction to Enthalpy= 0.463889
C -0.07634100 1.29673900 -0.19841600	Thermal correction to Gibbs Free Energy= 0.386121
C -1.11405200 2.39991700 -0.13157300	Sum of electronic and zero-point Energies= -1153.535316
C -2.50254900 1.93192800 -0.53958300	Sum of electronic and thermal Energies= -1153.511108
C 0.59445500 -1.02047200 -0.45152600	Sum of electronic and thermal Enthalpies= -1153.510164
C 1.26470700 1.70504200 -0.17850900	Sum of electronic and thermal Free Energies= -1153.587932
H -3.22828600 2.71507500 -0.30498100	
C 2.26864700 0.75185700 -0.27178900	
C 1.90112500 -0.57908500 -0.41126100	
H 1.50330300 2.76248800 -0.10083800	
O 2.98798500 -1.42088200 -0.57999900	
C 3.73653900 0.78732500 -0.29508400	
C -2.14204300 -1.72418900 0.65150700	
H -1.74467200 -1.44985400 1.63954700	
H -1.58496700 -2.59695300 0.31225500	
C -3.62036100 -2.09485300 0.79461600	
H -4.00860000 -2.46934500 -0.16058600	
H -3.70862600 -2.92732500 1.50211200	
C -4.46105900 -0.91975400 1.28146900	
H -4.14434700 -0.66295600 2.30347600	
H -5.52084400 -1.20153400 1.34133500	
C -4.32304200 0.34054100 0.40781900	
C -4.96461600 1.50336600 1.18598800	
H -5.10940000 2.39241800 0.56442200	
H -5.95264800 1.19939500 1.55083800	
H -4.35783400 1.78651500 2.05446400	

C -5.11113200 0.18577700 -0.90308800<br>H -6.18441000 0.26379400 -0.69276800<br>H -4.85797200 0.97767100 -1.61758000<br>H -4.94929100 -0.77553300 -1.39375500<br>C -2.22323600 -0.96046300 -1.76280600<br>H -2.26379800 -0.10390300 -2.44186400<br>H -1.43683300 -1.63070600 -2.12004000<br>H -3.16629700 -1.50101100 -1.84385700<br>H -1.15749500 2.78100000 0.89800100<br>H -0.77702500 3.23295800 -0.75776500<br>H -2.54653200 1.77379600 -1.62332900<br>H -2.44721200 0.80360700 1.24771400<br>C 4.11912200 -0.63846500 -0.58167200<br>O 5.21346100 -1.11493900 -0.73161600<br>O 0.33830200 -2.33901700 -0.67552300<br>H 1.17375900 -2.82019100 -0.76828700<br>C 4.52155600 1.88451600 -0.97368500<br>H 5.59450900 1.70603500 -0.87133100<br>H 4.27168300 1.92467900 -2.03925800<br>H 4.28295700 2.85281400 -0.52668900<br>H 4.15125200 0.81897900 0.91136100<br>O 4.92738100 -0.63079800 2.20766600<br>H 5.76353800 -0.87435500 1.76821600<br>O 4.81224100 0.71403800 1.99916600	
**Name**	**5-O12-H-OOH (gas phase)**
Cartesian Coordinates	Frequency and Energy
C -1.96874300 0.30555300 0.35252400<br>C -1.12247500 -0.50806400 -0.67521500<br>C 0.33632600 -0.06583300 -0.51452200<br>C 0.65739200 1.27940000 -0.20500200<br>C -0.41358300 2.29132800 0.04321900<br>C -1.84612000 1.80557500 0.07814400<br>H 1.23845000 -1.98244400 -0.97485900<br>C 1.39189800 -0.93818400 -0.72570900<br>C 1.98762300 1.71069900 -0.11932400<br>H -2.35885700 2.40895300 0.83147800<br>C 3.05110100 0.85014500 -0.33404400<br>C 2.73931500 -0.50711100 -0.65137300<br>H 2.16344300 2.75564300 0.12038100<br>O 3.69707900 -1.36809200 -0.90958000<br>H 4.31222300 -1.55260700 0.02457200<br>C 4.48265000 1.28184200 -0.25142300<br>H 4.97615200 0.81329900 0.60698300<br>H 5.02899600 0.97083000 -1.14657300<br>H 4.55318100 2.36563600 -0.14482500<br>O -0.14405200 3.46545600 0.21377000<br>C -1.27079800 -2.01282700 -0.37422700<br>H -0.76375300 -2.23523900 0.57563700<br>H -0.76657300 -2.59865600 -1.15061300<br>C -2.72825200 -2.45814700 -0.26708500<br>H -3.23672000 -2.33780400 -1.23133400<br>H -2.76223100 -3.52852000 -0.03816700<br>C -3.45375900 -1.67222000 0.82033500<br>H -2.96900800 -1.88085200 1.78577200	Zero-point correction= 0.398400 (Hartree/Particle)<br>Thermal correction to Energy= 0.420218<br>Thermal correction to Enthalpy= 0.421162<br>Thermal correction to Gibbs Free Energy= 0.347954<br>Sum of electronic and zero-point Energies= -1000.962147<br>Sum of electronic and thermal Energies= -1000.940329<br>Sum of electronic and thermal Enthalpies= -1000.939385<br>Sum of electronic and thermal Free Energies= -1001.012593

H -4.49401500 -2.00901900 0.91294500<br>C -3.44009100 -0.15079000 0.58614800<br>C -3.97017100 0.51944700 1.86574400<br>H -4.14816200 1.59032800 1.73038600<br>H -4.92509200 0.06535700 2.15180000<br>H -3.27323000 0.38971200 2.70123300<br>C -4.40750600 0.21501200 -0.55152100<br>H -5.43145700 -0.01652800 -0.23817300<br>H -4.37415900 1.28414700 -0.78305600<br>H -4.22034700 -0.33587100 -1.47446500<br>C -1.48330900 -0.24879200 -2.15545100<br>H -1.41186800 0.81009500 -2.41890700<br>H -0.77478800 -0.78807000 -2.79205900<br>H -2.48665700 -0.59472700 -2.40641500<br>H -2.29588900 2.06886600 -0.88726400<br>H -1.47021900 0.11797400 1.31880300<br>O 3.74986700 -0.92694200 1.87864000<br>H 3.09354000 -1.54939400 2.23434700<br>O 4.64061400 -1.72208500 1.21280400	
**Name**	**5-O12-H-OOH (water)**
Cartesian Coordinates	Frequency and Energy
C -1.93757000 0.30110700 0.35365700<br>C -1.09167000 -0.48021800 -0.69685200<br>C 0.36203500 -0.02985700 -0.52906600<br>C 0.67742500 1.30871900 -0.18019100<br>C -0.40573000 2.29814300 0.07840000<br>C -1.82594800 1.80741100 0.11590000<br>H 1.26734600 -1.92781900 -1.04355200<br>C 1.42266800 -0.89110500 -0.76467300<br>C 2.00595000 1.74200200 -0.06207800<br>H -2.33972900 2.38732400 0.88734600<br>C 3.07247600 0.88929900 -0.29082400<br>C 2.76329300 -0.45342100 -0.66330300<br>H 2.19588100 2.77306400 0.22097300<br>O 3.74470900 -1.30076700 -0.93603200<br>H 4.23354400 -1.60751300 0.03413900<br>C 4.50019000 1.31434000 -0.15938500<br>H 5.01598600 0.70230500 0.58914600<br>H 5.03303900 1.17837400 -1.10608300<br>H 4.56373300 2.36271200 0.13645100<br>O -0.14430100 3.48561800 0.25143900<br>C -1.22424600 -1.99239500 -0.42884700<br>H -0.71579300 -2.23026000 0.51622800<br>H -0.71679000 -2.55398100 -1.22051200<br>C -2.67884000 -2.44835600 -0.33069600<br>H -3.18798800 -2.30729900 -1.29185400<br>H -2.70307500 -3.52353800 -0.12325200<br>C -3.40901000 -1.69208000 0.77439300<br>H -2.92034700 -1.91637300 1.73412300<br>H -4.44821900 -2.03476000 0.85793500<br>C -3.40623800 -0.16645900 0.57280600<br>C -3.94079300 0.47350400 1.86465100<br>H -4.12720600 1.54595000 1.74959400<br>H -4.89232300 0.00419900 2.13900400	Zero-point correction= 0.397225 (Hartree/Particle)<br>Thermal correction to Energy= 0.418966<br>Thermal correction to Enthalpy= 0.419910<br>Thermal correction to Gibbs Free Energy= 0.347009<br>Sum of electronic and zero-point Energies= -1000.983719<br>Sum of electronic and thermal Energies= -1000.961978<br>Sum of electronic and thermal Enthalpies= -1000.961034<br>Sum of electronic and thermal Free Energies= -1001.033935

H -3.24103000 0.33203200 2.69630000	
C -4.37275500 0.21677700 -0.55865000	
H -5.39631000 -0.01652100 -0.24333500	
H -4.33546800 1.28898000 -0.77745800	
H -4.18830700 -0.32585300 -1.48750200	
C -1.45691000 -0.18904600 -2.16911200	
H -1.40133400 0.87746900 -2.40691100	
H -0.74374000 -0.70860200 -2.81778700	
H -2.45630200 -0.54424100 -2.42320700	
H -2.27722400 2.09366200 -0.84315100	
H -1.43940900 0.09189900 1.31449500	
O 3.47115100 -1.12002100 1.85913500	
H 2.71851400 -1.73576500 1.95327300	
O 4.45051500 -1.84678200 1.25030800	
**Name**	**5-O12-H-OOH (pentyl ethanoate)**
Cartesian Coordinates	Frequency and Energy
C -1.95885200 0.30195600 0.35460700	Zero-point correction= 0.397760 (Hartree/Particle)
C -1.11337000 -0.49580400 -0.68560500	Thermal correction to Energy= 0.419625
C 0.34311600 -0.04769900 -0.52417700	Thermal correction to Enthalpy= 0.420569
C 0.66098100 1.29467800 -0.19635300	Thermal correction to Gibbs Free Energy= 0.346753
C -0.41677000 2.29675700 0.06274500	Sum of electronic and zero-point Energies= -1000.988134
C -1.84312100 1.80561100 0.09820600	Sum of electronic and thermal Energies= -1000.966270
H 1.24541700 -1.95535100 -1.01007100	Sum of electronic and thermal Enthalpies= -1000.965326
C 1.40202500 -0.91452000 -0.74727300	Sum of electronic and thermal Free Energies= -1001.039141
C 1.99068800 1.72744600 -0.09906500	
H -2.35795500 2.39513500 0.86142600	
C 3.05647500 0.87122100 -0.32066500	
C 2.74658000 -0.48091000 -0.65991400	
H 2.17264000 2.76702400 0.15826300	
O 3.71316700 -1.33505100 -0.92618700	
H 4.28107500 -1.56696800 0.01684800	
C 4.48553800 1.30428500 -0.22153900	
H 5.00159400 0.76083600 0.57774800	
H 5.02067600 1.09040400 -1.15230300	
H 4.55063200 2.37360900 -0.01194200	
O -0.14924500 3.47418900 0.23869200	
C -1.25168500 -2.00468500 -0.40007500	
H -0.74144400 -2.23515800 0.54621500	
H -0.74777200 -2.57842400 -1.18565800	
C -2.70682600 -2.45721600 -0.29308300	
H -3.21778400 -2.32951000 -1.25526600	
H -2.73314000 -3.52992300 -0.07231300	
C -3.43435500 -1.68551300 0.80309600	
H -2.94722300 -1.90072000 1.76597900	
H -4.47367600 -2.02694400 0.89176700	
C -3.42862900 -0.16185400 0.58329700	
C -3.96151600 0.49442900 1.86825800	
H -4.15432600 1.56429800 1.74025000	
H -4.91008400 0.02626700 2.15499600	
H -3.26003900 0.36914000 2.70137700	
C -4.39695700 0.20884800 -0.55111800	
H -5.42118100 -0.02071200 -0.23494400	
H -4.36199800 1.27857900 -0.78217000	
H -4.21379900 -0.34255300 -1.47502700	

C -1.48012400 -0.22249700 -2.16119900<br>H -1.42774200 0.84102800 -2.41301200<br>H -0.76634600 -0.74498300 -2.80697500<br>H -2.47861600 -0.58247800 -2.41300100<br>H -2.29484200 2.07931700 -0.86390200<br>H -1.45973700 0.10424600 1.31794400<br>O 3.67931400 -1.00313300 1.88364700<br>H 2.97367900 -1.61726000 2.16003000<br>O 4.57711400 -1.78926600 1.21920700	
**Name**	**6-O12-H-OOH (gas phase)**
Cartesian Coordinates	Frequency and Energy
C -2.00911100 0.48262700 0.29681900<br>C -1.10513600 -0.44292000 -0.57236600<br>C 0.34673200 0.04642000 -0.44447200<br>C 0.65403900 1.39741300 -0.13041200<br>C -0.41995100 2.40658500 0.20850100<br>C -1.83245800 1.94120300 -0.13226000<br>H 1.22935300 -1.86606500 -0.95491200<br>C 1.39786600 -0.82451700 -0.70363500<br>C 1.98140900 1.83108100 -0.08651700<br>H -2.55015000 2.59110500 0.37584800<br>C 3.01854400 0.95386600 -0.35680000<br>C 2.73519600 -0.40155200 -0.67292100<br>H 2.21550600 2.86401100 0.15616300<br>O 3.75485900 -1.20352100 -0.94792600<br>H 4.27156700 -1.46478000 -0.00201200<br>C -1.24062600 -1.88772300 -0.04476200<br>H -0.78659800 -1.94184500 0.95458000<br>H -0.68239000 -2.57821900 -0.68556500<br>C -2.69533300 -2.35120200 0.03901200<br>H -3.13573100 -2.40255900 -0.96420600<br>H -2.72617800 -3.37201800 0.43479600<br>C -3.51663300 -1.42785100 0.93363100<br>H -3.11618700 -1.48690300 1.95664000<br>H -4.55926100 -1.76745200 0.98526200<br>C -3.49041700 0.04641700 0.48908100<br>C -4.10867800 0.87669000 1.62845300<br>H -4.30522500 1.91099200 1.33090100<br>H -5.06747800 0.43771700 1.92552400<br>H -3.45793600 0.88964300 2.51017400<br>C -4.37677100 0.24949300 -0.75116000<br>H -5.42960500 0.15467800 -0.46274300<br>H -4.24252200 1.24794100 -1.18082100<br>H -4.19230800 -0.48344100 -1.53801600<br>C -1.42555500 -0.42920400 -2.08432900<br>H -1.36018600 0.57537800 -2.51004600<br>H -0.68875500 -1.04762300 -2.60706600<br>H -2.41367600 -0.83071900 -2.30971700<br>H -2.01987000 2.05194800 -1.20547900<br>H -1.57555800 0.40388400 1.30995700<br>O 4.30662700 1.35440100 -0.27579900<br>H 4.86193900 0.64279800 -0.63728500<br>H -0.36453400 2.59809700 1.28968400<br>H -0.18792600 3.35846200 -0.28192600	Zero-point correction= 0.394961 (Hartree/Particle)<br>Thermal correction to Energy= 0.415330<br>Thermal correction to Enthalpy= 0.416274<br>Thermal correction to Gibbs Free Energy= 0.346558<br>Sum of electronic and zero-point Energies= -962.873505<br>Sum of electronic and thermal Energies= -962.853136<br>Sum of electronic and thermal Enthalpies= -962.852192<br>Sum of electronic and thermal Free Energies= -962.921907

O 3.77908200 -0.69096900 1.84521700<br>H 4.12381400 0.22086400 1.86686700<br>O 4.74458000 -1.39826600 1.19703600	
**Name**	**6-O12-H-OOH (water)**
Cartesian Coordinates	Frequency and Energy
C -1.98673000 0.46840900 0.32021500<br>C -1.07981700 -0.39965500 -0.60310500<br>C 0.36773100 0.09018800 -0.44845900<br>C 0.66393600 1.43114400 -0.07683400<br>C -0.41643200 2.41179700 0.31442000<br>C -1.82833100 1.94935700 -0.03124700<br>H 1.26465300 -1.79095800 -1.03791800<br>C 1.42798700 -0.75979300 -0.74118600<br>C 1.98741800 1.87425200 -0.02161000<br>H -2.54294500 2.56239900 0.52454100<br>C 3.03567600 1.01812300 -0.32393000<br>C 2.75948900 -0.32537600 -0.68247000<br>H 2.20871200 2.89976400 0.26211800<br>O 3.79071900 -1.13119500 -0.98767500<br>H 4.25446000 -1.44825100 -0.08119400<br>C -1.20595900 -1.87522000 -0.16901700<br>H -0.75923900 -1.98891200 0.82905100<br>H -0.64027400 -2.51819700 -0.85129800<br>C -2.65853600 -2.34851200 -0.11933100<br>H -3.09708700 -2.32691400 -1.12452200<br>H -2.68539400 -3.39447600 0.20541900<br>C -3.48053000 -1.49119400 0.83774900<br>H -3.07341800 -1.61453200 1.85242300<br>H -4.52223100 -1.83649900 0.86945000<br>C -3.46335100 0.00847600 0.49041400<br>C -4.07892300 0.75973000 1.68329900<br>H -4.27332500 1.81250000 1.45602400<br>H -5.03783200 0.30092900 1.95054200<br>H -3.42466700 0.71248400 2.56161200<br>C -4.35792800 0.28418700 -0.72885100<br>H -5.40835000 0.16844600 -0.43704100<br>H -4.22906300 1.30776600 -1.09752100<br>H -4.17777200 -0.40073900 -1.55963700<br>C -1.39891500 -0.29174200 -2.11095700<br>H -1.31825300 0.73645000 -2.47451400<br>H -0.67262000 -0.89363200 -2.66820300<br>H -2.39389000 -0.66354200 -2.35803200<br>H -2.03342400 2.11592200 -1.09387200<br>H -1.55041800 0.33803100 1.32643100<br>O 4.31656600 1.45276600 -0.25574000<br>H 4.90828100 0.74380400 -0.56189600<br>H -0.34777500 2.55241400 1.40228100<br>H -0.19426300 3.38632800 -0.13319500<br>O 3.58134500 -0.89993900 1.83511200<br>H 3.96686700 -0.01795500 2.00607900<br>O 4.56869100 -1.61759000 1.24558600	Zero-point correction= 0.393242 (Hartree/Particle)<br>Thermal correction to Energy= 0.413795<br>Thermal correction to Enthalpy= 0.414739<br>Thermal correction to Gibbs Free Energy= 0.344765<br>Sum of electronic and zero-point Energies= -962.893320<br>Sum of electronic and thermal Energies= -962.872767<br>Sum of electronic and thermal Enthalpies= -962.871823<br>Sum of electronic and thermal Free Energies= -962.941797
**Name**	**6-O12-H-OOH (pentyl ethanoate)**
Cartesian Coordinates	Frequency and Energy
C -2.00716700 0.47895100 0.30579900	Zero-point correction= 0.394159 (Hartree/Particle)

C -1.10266800 -0.43613600 -0.57419000	Thermal correction to Energy= 0.414603
C 0.34895400 0.05218900 -0.44108800	Thermal correction to Enthalpy= 0.415547
C 0.65533600 1.40263700 -0.11731700	Thermal correction to Gibbs Free Energy= 0.345831
C -0.41844600 2.40587200 0.23481800	Sum of electronic and zero-point Energies= -962.896524
C -1.83177000 1.94277100 -0.10480800	Sum of electronic and thermal Energies= -962.876081
H 1.23036800 -1.85588100 -0.96590500	Sum of electronic and thermal Enthalpies= -962.875137
C 1.40154000 -0.81581600 -0.70792900	Sum of electronic and thermal Free Energies= -962.944852
C 1.98250300 1.83865700 -0.07847500	
H -2.54571400 2.58534600 0.41772200	
C 3.02072000 0.96501600 -0.35936900	
C 2.73678300 -0.38908500 -0.67507200	
H 2.21233700 2.87188500 0.16842000	
O 3.75969000 -1.19491500 -0.95783100	
H 4.26491800 -1.45620500 -0.03615400	
C -1.23846200 -1.88840300 -0.06775800	
H -0.79018900 -1.95688900 0.93361100	
H -0.67783700 -2.56878700 -0.71746800	
C -2.69374700 -2.35171400 0.00456400	
H -3.13012200 -2.38640100 -1.00123200	
H -2.72549500 -3.37835400 0.38622100	
C -3.51763000 -1.44145400 0.91002700	
H -3.12071300 -1.51627800 1.93353300	
H -4.56111600 -1.78075800 0.95100300	
C -3.48915100 0.03951300 0.48849800	
C -4.10783500 0.85270700 1.63921000	
H -4.30401100 1.89205200 1.35740200	
H -5.06736100 0.40940800 1.92973600	
H -3.45739800 0.85326800 2.52186600	
C -4.37408800 0.26254000 -0.74894500	
H -5.42789900 0.16764600 -0.46143300	
H -4.23742600 1.26677600 -1.16532000	
H -4.19394800 -0.46028000 -1.54694700	
C -1.42103100 -0.40209300 -2.08590800	
H -1.35689800 0.60838100 -2.49916700	
H -0.68509700 -1.01582200 -2.61686700	
H -2.40897800 -0.80208900 -2.31642300	
H -2.02508400 2.06820000 -1.17544600	
H -1.57699300 0.38684400 1.31890900	
O 4.30642200 1.37562500 -0.29358300	
H 4.86966400 0.68201900 -0.67780700	
H -0.35668600 2.58554400 1.31767700	
H -0.18773700 3.36252700 -0.24669200	
O 3.76579200 -0.72119200 1.85385200	
H 4.12246900 0.18752900 1.89533500	
O 4.72733500 -1.44034300 1.21794600	
**Name**	**6-O12-H-OOH (6-311++G(d,); pentyl ethanoate)**
Cartesian Coordinates	Frequency and Energy
C -2.00175700 0.47826100 0.30844500	Zero-point correction= 0.393784 (Hartree/Particle)
C -1.10085900 -0.43342300 -0.57705000	Thermal correction to Energy= 0.414221
C 0.35068700 0.05155000 -0.44245300	Thermal correction to Enthalpy= 0.415166
C 0.65776000 1.39969800 -0.11973500	Thermal correction to Gibbs Free Energy= 0.345425
C -0.41341400 2.40353600 0.23376100	Sum of electronic and zero-point Energies= -963.119410
C -1.82821400 1.94223100 -0.09912500	Sum of electronic and thermal Energies= -963.098973
H 1.22943000 -1.85372800 -0.96433900	Sum of electronic and thermal Enthalpies= -963.098029

C 1.40051300 -0.81555800 -0.70702300<br>C 1.98298400 1.83340500 -0.08223600<br>H -2.53681200 2.58221000 0.42920300<br>C 3.01946300 0.96101400 -0.36038900<br>C 2.73455100 -0.39146500 -0.67567700<br>H 2.21361300 2.86479600 0.16362600<br>O 3.75284100 -1.19878000 -0.95561200<br>H 4.26512700 -1.44453300 -0.03523100<br>C -1.23771200 -1.88725800 -0.07840800<br>H -0.78934700 -1.96052300 0.92007000<br>H -0.68090000 -2.56341800 -0.73187100<br>C -2.69314200 -2.34795400 -0.00685200<br>H -3.13016500 -2.37575100 -1.00996700<br>H -2.72563300 -3.37478000 0.36841700<br>C -3.51250200 -1.44227600 0.90606000<br>H -3.11275500 -1.52185900 1.92558600<br>H -4.55435900 -1.77914800 0.94783700<br>C -3.48302100 0.03936000 0.49062600<br>C -4.09856600 0.84823800 1.64515200<br>H -4.29824900 1.88552900 1.36594200<br>H -5.05414700 0.40211500 1.93723800<br>H -3.44555900 0.84794500 2.52342000<br>C -4.36989200 0.26705400 -0.74357100<br>H -5.42126500 0.18133000 -0.45117400<br>H -4.22753600 1.26772700 -1.16140000<br>H -4.19985500 -0.45846700 -1.53850300<br>C -1.41740100 -0.38954000 -2.08783000<br>H -1.33946200 0.62001100 -2.49578700<br>H -0.68826700 -1.00861000 -2.61841600<br>H -2.40763200 -0.77658400 -2.32073500<br>H -2.02751400 2.07020300 -1.16580500<br>H -1.57014900 0.38224500 1.31766700<br>O 4.30304000 1.37279800 -0.29627400<br>H 4.86566800 0.67667900 -0.66837200<br>H -0.34593800 2.58372700 1.31420800<br>H -0.18274200 3.35719600 -0.24917200<br>O 3.74353900 -0.73862400 1.86477600<br>H 4.07263200 0.17550300 1.92947500<br>O 4.71994900 -1.42052500 1.21784800	Sum of electronic and thermal Free Energies= -963.167770
**Name**	**6-O13-H-OOH (gas phase)**
Cartesian Coordinates	Frequency and Energy
C -2.00003300 0.54955400 0.22814000<br>C -1.15388300 -0.54687000 -0.48729900<br>C 0.32164000 -0.12107100 -0.45000300<br>C 0.70011700 1.24504100 -0.34248200<br>C -0.31666700 2.35127400 -0.15150800<br>C -1.75236200 1.91266400 -0.42252100<br>H 1.07330100 -2.14375600 -0.69914300<br>C 1.31708000 -1.09198400 -0.59929200<br>C 2.04348500 1.58978600 -0.37100000<br>H -2.43510600 2.66813600 -0.02418900<br>C 3.04679300 0.62331700 -0.52509900<br>C 2.65731500 -0.73779700 -0.63968000<br>H 2.35298400 2.62769200 -0.28150600	Zero-point correction= 0.395023 (Hartree/Particle)<br>Thermal correction to Energy= 0.415400<br>Thermal correction to Enthalpy= 0.416344<br>Thermal correction to Gibbs Free Energy= 0.346630<br>Sum of electronic and zero-point Energies= -962.873070<br>Sum of electronic and thermal Energies= -962.852694<br>Sum of electronic and thermal Enthalpies= -962.851749<br>Sum of electronic and thermal Free Energies= -962.921463

O	3.61441800	-1.68731200	-0.73994100	
H	4.46368100	-1.23897400	-0.89157900	
C	-1.35796600	-1.88243000	0.26154200	
H	-0.89862100	-1.80180000	1.25683100	
H	-0.84331700	-2.69416800	-0.26295500	
C	-2.83384600	-2.25006900	0.41975700	
H	-3.28539200	-2.43465800	-0.56259300	
H	-2.91291400	-3.19366900	0.97034500	
C	-3.59789700	-1.15658600	1.15966500	
H	-3.19160200	-1.07554400	2.17886000	
H	-4.65594500	-1.42942200	1.26491500	
C	-3.49984800	0.22603000	0.48879000	
C	-4.06315500	1.25603800	1.48459300	
H	-4.21180900	2.23870500	1.02747600	
H	-5.03894800	0.91844600	1.85083200	
H	-3.40229700	1.37570000	2.35056300	
C	-4.38726700	0.27791900	-0.76637200	
H	-5.44040800	0.29372500	-0.46438400	
H	-4.20002100	1.18356800	-1.35307000	
H	-4.25559800	-0.58275300	-1.42401200	
C	-1.49053400	-0.75339400	-1.98261200	
H	-1.37337300	0.16634200	-2.56098900	
H	-0.79942600	-1.48987500	-2.40493200	
H	-2.50384800	-1.12484300	-2.13500900	
H	-1.93845300	1.86403400	-1.50070900	
H	-1.56122900	0.60692000	1.24040900	
O	4.33897800	0.91140500	-0.58662500	
H	4.81720500	0.61650700	0.37180100	
H	-0.24688600	2.69778800	0.88883600	
H	-0.04250600	3.20544200	-0.77974800	
O	3.83994000	-0.33434300	1.91706300	
H	3.72648900	-1.26538400	1.65051200	
O	5.08244100	-0.00683700	1.46771900	
**Name**				**6-O13-H-OOH (water)**
Cartesian Coordinates				Frequency and Energy
C	-1.98964600	0.56829800	0.19780800	Zero-point correction= 0.393560 (Hartree/Particle)
C	-1.15670900	-0.57818300	-0.44946300	Thermal correction to Energy= 0.413959
C	0.32208500	-0.16594900	-0.44734400	Thermal correction to Enthalpy= 0.414903
C	0.71666000	1.20120700	-0.40004900	Thermal correction to Gibbs Free Energy= 0.345611
C	-0.28641100	2.32601500	-0.26379700	Sum of electronic and zero-point Energies= -962.892605
C	-1.72312900	1.88879600	-0.52820500	Sum of electronic and thermal Energies= -962.872206
H	1.04373300	-2.20400300	-0.63451800	Sum of electronic and thermal Enthalpies= -962.871261
C	1.30478400	-1.15294000	-0.56971300	Sum of electronic and thermal Free Energies= -962.940553
C	2.06545200	1.52562100	-0.45044500	
H	-2.40034600	2.67300500	-0.17949100	
C	3.05202800	0.53912900	-0.56683700	
C	2.65137600	-0.81994400	-0.63383200	
H	2.38361300	2.56442200	-0.40816000	
O	3.58415300	-1.79580600	-0.74626800	
H	4.46040200	-1.38860500	-0.85917200	
C	-1.36615100	-1.86141500	0.38443900	
H	-0.91053200	-1.71580700	1.37413100	
H	-0.85469300	-2.70671700	-0.08685000	
C	-2.84510100	-2.20654900	0.56313000	

H -3.29564800 -2.44912400 -0.40704800<br>H -2.93024900 -3.11060800 1.17604600<br>C -3.60364600 -1.06153100 1.22708900<br>H -3.19903800 -0.91532700 2.23970200<br>H -4.66490200 -1.31785100 1.34297000<br>C -3.49251000 0.27315100 0.46790100<br>C -4.05312400 1.37027100 1.38878800<br>H -4.18662800 2.32223700 0.86571900<br>H -5.03549400 1.06474900 1.76692300<br>H -3.39619600 1.53987300 2.24991800<br>C -4.37060600 0.24835400 -0.79370800<br>H -5.42499800 0.30901600 -0.49975300<br>H -4.15953800 1.10348000 -1.44558700<br>H -4.25267200 -0.66353900 -1.38205800<br>C -1.50149600 -0.87684500 -1.92656700<br>H -1.39457900 0.00787500 -2.56015500<br>H -0.81241900 -1.63900600 -2.30564600<br>H -2.51440800 -1.26170200 -2.04597000<br>H -1.89571500 1.77970600 -1.60430800<br>H -1.55681300 0.67851400 1.20794000<br>O 4.35976800 0.82893900 -0.65359300<br>H 4.82390800 0.60043300 0.28210900<br>H -0.22122300 2.71451400 0.76163500<br>H 0.00658500 3.14798700 -0.92516100<br>O 3.79483400 -0.12144300 1.96970900<br>H 3.69572600 -1.08597500 1.84477600<br>O 5.05486800 0.16925300 1.55975800	
**Name**	**6-O13-H-OOH (pentyl ethanoate)**
Cartesian Coordinates	Frequency and Energy
C -1.99614600 0.55466200 0.22357400<br>C -1.15429000 -0.55344200 -0.47859800<br>C 0.32204500 -0.13017500 -0.45296700<br>C 0.70438400 1.23742800 -0.35740700<br>C -0.30912700 2.34757700 -0.17772700<br>C -1.74534000 1.90879500 -0.44400400<br>H 1.06424600 -2.15574600 -0.69538100<br>C 1.31471400 -1.10466600 -0.60022500<br>C 2.04962100 1.57760400 -0.39122400<br>H -2.42493600 2.67100400 -0.05311600<br>C 3.04721400 0.60547500 -0.53660100<br>C 2.65659600 -0.75453800 -0.64710400<br>H 2.35865800 2.61686800 -0.31008900<br>O 3.60756600 -1.70857600 -0.75725700<br>H 4.45931000 -1.27394000 -0.93471700<br>C -1.35735400 -1.87898600 0.28863400<br>H -0.89748400 -1.78535800 1.28273500<br>H -0.84561400 -2.69808200 -0.22740000<br>C -2.83391500 -2.24062100 0.45450700<br>H -3.28666000 -2.43669700 -0.52519500<br>H -2.91270200 -3.17585800 1.02002700<br>C -3.59639300 -1.13542200 1.17853100<br>H -3.18987400 -1.03857300 2.19639900<br>H -4.65528000 -1.40532000 1.28597000	Zero-point correction= 0.394318 (Hartree/Particle)<br>Thermal correction to Energy= 0.414692<br>Thermal correction to Enthalpy= 0.415636<br>Thermal correction to Gibbs Free Energy= 0.346164<br>Sum of electronic and zero-point Energies= -962.896011<br>Sum of electronic and thermal Energies= -962.875636<br>Sum of electronic and thermal Enthalpies= -962.874692<br>Sum of electronic and thermal Free Energies= -962.944165

C -3.49674400 0.23623600 0.48588900<br>C -4.06072200 1.28343500 1.46209600<br>H -4.20981400 2.25800000 0.98675800<br>H -5.03677300 0.95179300 1.83485500<br>H -3.39972200 1.42058800 2.32605200<br>C -4.38089500 0.26625100 -0.77184300<br>H -5.43495200 0.30359400 -0.47254900<br>H -4.18185400 1.15325800 -1.38364000<br>H -4.25922100 -0.61472800 -1.40497100<br>C -1.49681900 -0.77928100 -1.96923700<br>H -1.39180300 0.13503400 -2.55956000<br>H -0.80487500 -1.51796000 -2.38786300<br>H -2.50853900 -1.16065300 -2.10964200<br>H -1.93061700 1.84758000 -1.52174200<br>H -1.55958100 0.62315900 1.23584000<br>O 4.34558900 0.89383200 -0.59973800<br>H 4.80924800 0.61726600 0.34171200<br>H -0.23720500 2.70401400 0.85911600<br>H -0.03125400 3.19404300 -0.81480600<br>O 3.82343200 -0.29979300 1.93601600<br>H 3.72602400 -1.24249700 1.69842100<br>O 5.07095200 0.03544000 1.51285700	
**Name**	**6-O13-H-OOH (6-311++G(d,); pentyl ethanoate)**
Cartesian Coordinates	Frequency and Energy
C -1.99121400 0.55364100 0.22562100<br>C -1.15099000 -0.55136400 -0.48126400<br>C 0.32425300 -0.12864600 -0.45426300<br>C 0.70533500 1.23702600 -0.35868400<br>C -0.30735800 2.34672200 -0.17917000<br>C -1.74396500 1.90834100 -0.44010700<br>H 1.06696200 -2.14977700 -0.69619600<br>C 1.31592800 -1.10040500 -0.60090200<br>C 2.04761700 1.57640200 -0.39080700<br>H -2.42002400 2.66799500 -0.04433000<br>C 3.04517600 0.60741800 -0.53647700<br>C 2.65555700 -0.75139500 -0.64718000<br>H 2.35591800 2.61389800 -0.30880600<br>O 3.60382400 -1.70550400 -0.75916200<br>H 4.45570700 -1.27283200 -0.92238300<br>C -1.35308700 -1.87934400 0.28021100<br>H -0.89378600 -1.78902600 1.27235200<br>H -0.84351300 -2.69447100 -0.23932800<br>C -2.82911300 -2.24087600 0.44512200<br>H -3.28156600 -2.43235000 -0.53293300<br>H -2.90715700 -3.17658700 1.00604300<br>C -3.58947300 -1.13925100 1.17530200<br>H -3.18113400 -1.04604000 2.19025700<br>H -4.64619200 -1.40838800 1.28282900<br>C -3.49066000 0.23363300 0.48715000<br>C -4.05344700 1.27716400 1.46693000<br>H -4.20715500 2.24983500 0.99375900<br>H -5.02537900 0.94188200 1.84161600<br>H -3.39047700 1.41404600 2.32688600<br>C -4.37603000 0.26700700 -0.76868700	Zero-point correction= 0.393851 (Hartree/Particle)<br>Thermal correction to Energy= 0.414248<br>Thermal correction to Enthalpy= 0.415192<br>Thermal correction to Gibbs Free Energy= 0.345672<br>Sum of electronic and zero-point Energies= -963.118960<br>Sum of electronic and thermal Energies= -963.098564<br>Sum of electronic and thermal Enthalpies= -963.097620<br>Sum of electronic and thermal Free Energies= -963.167140

H	-5.42718500	0.31220300	-0.46685800	
H	-4.17294700	1.15024100	-1.38108400	
H	-4.26281400	-0.61495400	-1.39841700	
C	-1.49090600	-0.77035300	-1.97248200	
H	-1.37505800	0.14294100	-2.55870900	
H	-0.80395500	-1.51233100	-2.38897700	
H	-2.50318000	-1.14277600	-2.11655000	
H	-1.93417800	1.84920200	-1.51457700	
H	-1.55386900	0.61907700	1.23484000	
O	4.33990500	0.89786400	-0.59493500	
H	4.80251200	0.60049400	0.33912000	
H	-0.23122700	2.70381300	0.85496800	
H	-0.03056500	3.18971300	-0.81781300	
O	3.80702500	-0.28693600	1.95031500	
H	3.67664700	-1.22599000	1.72835900	
O	5.05273100	0.01156000	1.50554100	

**Name**	**7-O12-H-OOH (gas phase)**
Cartesian Coordinates	Frequency and Energy
C -4.91656400 -1.69360600 0.06634700	Zero-point correction= 0.735095 (Hartree/Particle)
C -5.03035700 -0.14856600 0.23490200	Thermal correction to Energy= 0.772081
C -3.67830200 0.47769200 -0.14162800	Thermal correction to Enthalpy= 0.773026
C -2.46489300 -0.25117300 -0.04462400	Thermal correction to Gibbs Free Energy= 0.667439
C -2.43392300 -1.71390300 0.33456500	Sum of electronic and zero-point Energies= -1772.523489
C -3.75251200 -2.22924300 0.90372600	Sum of electronic and thermal Energies= -1772.486503
H -4.49556100 2.44256200 -0.54878600	Sum of electronic and thermal Enthalpies= -1772.485559
C -3.61111300 1.81662500 -0.50090600	Sum of electronic and thermal Free Energies= -1772.591146
C -1.24038500 0.36128600 -0.33889800	
H -3.73458200 -3.32248400 0.89993200	
C -1.19430400 1.68522400 -0.74398600	
C -2.39580300 2.45242900 -0.81289300	
H -0.32416700 -0.21632800 -0.26078100	
O -2.34612600 3.71818100 -1.15686500	
H -1.70144200 4.23474300 -0.44832600	
C -6.14345300 0.36024100 -0.70654500	
H -5.81265800 0.22768200 -1.74627200	
H -6.30361100 1.43321800 -0.55830200	
C -7.46773900 -0.37697700 -0.50423000	
H -7.86574000 -0.17345100 0.49733100	
H -8.20958500 0.01385800 -1.20890800	
C -7.29993400 -1.87889000 -0.71485800	
H -7.01085400 -2.05399700 -1.76189100	
H -8.25474400 -2.39870400 -0.56257800	
C -6.23157700 -2.51551300 0.19381200	
C -5.97901100 -3.94098900 -0.32945000	
H -5.37610500 -4.53741500 0.36167100	
H -6.93468500 -4.46128000 -0.45690500	
H -5.47323300 -3.92478500 -1.30155900	
C -6.75944800 -2.64802000 1.63212800	
H -7.52557400 -3.43067300 1.66540700	
H -5.96369700 -2.93763400 2.32671500	
H -7.21883900 -1.73298600 2.00882000	
C -5.33076600 0.32894700 1.67388200	
H -4.58267600 -0.02327600 2.38870900	
H -5.30514800 1.42305300 1.69710200	

H	-6.31275400	0.01301800	2.02587100	
H	-3.85873900	-1.92430000	1.95016800	
H	-4.62235900	-1.82614300	-0.99044800	
O	-0.07060100	2.37439900	-1.08054600	
H	-2.19313600	-2.28682300	-0.57246000	
H	-1.60639600	-1.88832100	1.03109700	
C	5.04630800	-1.30693900	-0.10060600	
C	5.07247600	0.22487600	0.18960700	
C	3.67134600	0.77080500	-0.12479900	
C	2.98372200	0.31241300	-1.26583500	
C	3.58035800	-0.74946100	-2.12256900	
C	4.64451200	-1.57573600	-1.54230700	
H	3.57679600	2.17926800	1.52684100	
C	3.08391600	1.77084700	0.65190600	
C	1.73774900	0.84222100	-1.59716000	
C	1.16158100	1.81003700	-0.79160400	
C	1.83563500	2.30007900	0.32686400	
H	1.19974800	0.49301500	-2.47494500	
O	1.28812300	3.25690500	1.11775300	
H	0.64929000	3.81954400	0.62396300	
C	5.43268300	0.43805600	1.67259600	
H	4.58792400	0.10435500	2.29151100	
H	5.57161200	1.50743400	1.87045900	
C	6.68628300	-0.32470100	2.10552300	
H	7.57096300	0.07453500	1.59518000	
H	6.85479700	-0.15672000	3.17454600	
C	6.54656800	-1.82187600	1.83418800	
H	5.71193700	-2.21232800	2.43470900	
H	7.44707900	-2.35841000	2.15966300	
C	6.27443200	-2.14128200	0.35388900	
C	5.89760600	-3.62586400	0.23021800	
H	5.73990000	-3.91882000	-0.81352600	
H	6.69776500	-4.25443600	0.63613000	
H	4.97692800	-3.84579700	0.78097100	
C	7.55489800	-1.91426900	-0.46883800	
H	8.29254700	-2.67741000	-0.19746600	
H	7.37919400	-2.00376500	-1.54527800	
H	8.01502700	-0.94215400	-0.28833700	
C	6.05581800	1.01002400	-0.70587200	
H	5.93203100	0.74914800	-1.76191100	
H	5.86185600	2.08238600	-0.60401400	
H	7.09682000	0.83380400	-0.43127600	
H	5.39030000	-1.99583700	-2.21483500	
H	4.20826200	-1.68822700	0.50180400	
H	3.54533500	-0.58761400	-3.19910800	
O	3.33690000	-2.10692600	-1.74014000	
O	-1.04393100	4.52516400	0.68366300	
O	-1.52628500	3.54621100	1.51216000	
H	-0.73932700	3.00826600	1.73183300	
**Name**				**7-O12-H-OOH (water)**
Cartesian Coordinates				Frequency and Energy
C	-4.86551501	-1.66197044	0.13849986	Zero-point correction= 0.735095 (Hartree/Particle)
C	-4.97930801	-0.11693044	0.30705486	Thermal correction to Energy= 0.771281

C	-3.62725301	0.50932756	-0.06947514	Thermal correction to Enthalpy= 0.771126
C	-2.41384401	-0.21953744	0.02752886	Thermal correction to Gibbs Free Energy= 0.665939
C	-2.38287401	-1.68226744	0.40671786	Sum of electronic and zero-point Energies= -1772.521729
C	-3.70146301	-2.19760744	0.97587886	Sum of electronic and thermal Energies= -1772.485967
H	-4.44451201	2.47419756	-0.47663314	Sum of electronic and thermal Enthalpies= -1772.483986
C	-3.56006401	1.84826056	-0.42875314	Sum of electronic and thermal Free Energies= -1772.599076
C	-1.18933601	0.39292156	-0.26674514	
H	-3.68353301	-3.29084844	0.97208486	
C	-1.14325501	1.71685956	-0.67183314	
C	-2.34475401	2.48406456	-0.74074014	
H	-0.27311801	-0.18469244	-0.18862814	
O	-2.29507701	3.74981656	-1.08471214	
H	-1.70144200	4.23474300	-0.44832600	
C	-6.09240401	0.39187656	-0.63439214	
H	-5.76160901	0.25931756	-1.67411914	
H	-6.25256201	1.46485356	-0.48614914	
C	-7.41669001	-0.34534144	-0.43207714	
H	-7.81469101	-0.14181544	0.56948386	
H	-8.15853601	0.04549356	-1.13675514	
C	-7.24888501	-1.84725444	-0.64270514	
H	-6.95980501	-2.02236144	-1.68973814	
H	-8.20369501	-2.36706844	-0.49042514	
C	-6.18052801	-2.48387744	0.26596486	
C	-5.92796201	-3.90935344	-0.25729714	
H	-5.32505601	-4.50577944	0.43382386	
H	-6.88363601	-4.42964444	-0.38475214	
H	-5.42218401	-3.89314944	-1.22940614	
C	-6.70839901	-2.61638444	1.70428086	
H	-7.47452501	-3.39903744	1.73755986	
H	-5.91264801	-2.90599844	2.39886786	
H	-7.16779001	-1.70135044	2.08097286	
C	-5.27971701	0.36058256	1.74603486	
H	-4.53162701	0.00835956	2.46086186	
H	-5.25409901	1.45468856	1.76925486	
H	-6.26170501	0.04465356	2.09802386	
H	-3.80769001	-1.89266444	2.02232086	
H	-4.57131001	-1.79450744	-0.91829514	
O	-0.01955201	2.40603456	-1.00839314	
H	-2.14208701	-2.25518744	-0.50030714	
H	-1.55534701	-1.85668544	1.10324986	
C	5.09735699	-1.27530344	-0.02845314	
C	5.12352499	0.25651156	0.26175986	
C	3.72239499	0.80244056	-0.05264614	
C	3.03477099	0.34404856	-1.19368214	
C	3.63140699	-0.71782544	-2.05041614	
C	4.69556099	-1.54410044	-1.47015414	
H	3.62784499	2.21090356	1.59899386	
C	3.13496499	1.80248256	0.72405886	
C	1.78879799	0.87385656	-1.52500714	
C	1.21262999	1.84167256	-0.71945114	
C	1.88668399	2.33171456	0.39901686	
H	1.25079699	0.52465056	-2.40279214	
O	1.33917199	3.28854056	1.18990586	
H	0.70033899	3.85117956	0.69611586	
C	5.48373199	0.46969156	1.74474886	

H 4.63897299 0.13599056 2.36366386	
H 5.62266099 1.53906956 1.94261186	
C 6.73733199 -0.29306544 2.17767586	
H 7.62201199 0.10617056 1.66733286	
H 6.90584599 -0.12508444 3.24669886	
C 6.59761699 -1.79024044 1.90634086	
H 5.76298599 -2.18069244 2.50686186	
H 7.49812799 -2.32677444 2.23181586	
C 6.32548099 -2.10964644 0.42604186	
C 5.94865499 -3.59422844 0.30237086	
H 5.79094899 -3.88718444 -0.74137314	
H 6.74881399 -4.22280044 0.70828286	
H 5.02797699 -3.81416144 0.85312386	
C 7.60594699 -1.88263344 -0.39668514	
H 8.34359599 -2.64577444 -0.12531314	
H 7.43024299 -1.97212944 -1.47312514	
H 8.06607599 -0.91051844 -0.21618414	
C 6.10686699 1.04165956 -0.63371914	
H 5.98307999 0.78078356 -1.68975814	
H 5.91290499 2.11402156 -0.53186114	
H 7.14786899 0.86543956 -0.35912314	
H 5.44134899 -1.96420144 -2.14268214	
H 4.25931099 -1.65659144 0.57395686	
H 3.59638399 -0.55597844 -3.12695514	
O 3.38794899 -2.07529044 -1.66798714	
O -1.04393100 4.52516400 0.68366300	
O -1.52628500 3.54621100 1.51216000	
H -0.73932700 3.00826600 1.73183300	
**Name**	**7-O12-H-OOH (pentyl ethanoate)**
Cartesian Coordinates	Frequency and Energy
C -4.93774900 -1.68825400 0.09359400	Zero-point correction= 0.734184 (Hartree/Particle)
C -5.06165800 -0.13943900 0.21297200	Thermal correction to Energy= 0.771208
C -3.69858000 0.48099800 -0.13217000	Thermal correction to Enthalpy= 0.772152
C -2.48655700 -0.24537700 0.01361400	Thermal correction to Gibbs Free Energy= 0.666485
C -2.46513300 -1.69508800 0.43618400	Sum of electronic and zero-point Energies= -1772.559627
C -3.79902600 -2.19502400 0.98176400	Sum of electronic and thermal Energies= -1772.522604
H -4.50991700 2.43366000 -0.59680100	Sum of electronic and thermal Enthalpies= -1772.521659
C -3.62295800 1.81357200 -0.51724700	Sum of electronic and thermal Free Energies= -1772.627326
C -1.25551400 0.35970900 -0.26862900	
H -3.77809100 -3.28781600 1.01002900	
C -1.20145600 1.67351500 -0.70473000	
C -2.40005500 2.43785800 -0.81011900	
H -0.34283900 -0.21772600 -0.15590000	
O -2.33989100 3.70380500 -1.18456500	
H -1.69236500 4.21233500 -0.51571900	
C -6.14017400 0.33847100 -0.78393200	
H -5.77268700 0.17215400 -1.80655800	
H -6.30658800 1.41540500 -0.67474700	
C -7.46936700 -0.39648300 -0.60655500	
H -7.90326000 -0.16283100 0.37340700	
H -8.18440100 -0.03056100 -1.35185200	
C -7.29250500 -1.90422600 -0.76116900	
H -6.96644900 -2.11427400 -1.79089600	
H -8.25188200 -2.42007300 -0.62286600	

C	-6.25591200	-2.50740400	0.20535600	
C	-5.98432500	-3.94955300	-0.25747900	
H	-5.41120300	-4.52208700	0.47856900	
H	-6.93529600	-4.47409200	-0.40626300	
H	-5.43797800	-3.96811500	-1.20794200	
C	-6.83296500	-2.58939300	1.62797300	
H	-7.59330300	-3.37844700	1.66511300	
H	-6.06019000	-2.84397500	2.36200000	
H	-7.31417400	-1.66474600	1.95171800	
C	-5.41686600	0.37670800	1.62547400	
H	-4.70132400	0.03850400	2.38027700	
H	-5.39051600	1.47177700	1.62258800	
H	-6.41477100	0.07369600	1.94337000	
H	-3.93588700	-1.85791500	2.01474700	
H	-4.61218700	-1.85225600	-0.94912600	
O	-0.07286700	2.35556500	-1.04191500	
H	-2.19696300	-2.29223300	-0.44720500	
H	-1.65715100	-1.84499000	1.16083300	
C	5.06459800	-1.31748000	-0.08949400	
C	5.09384500	0.22158500	0.15869400	
C	3.68381300	0.75750200	-0.13131700	
C	2.96518900	0.26874100	-1.24113800	
C	3.54139400	-0.81522900	-2.08450100	
C	4.62030500	-1.62540500	-1.51063500	
H	3.63872400	2.21011300	1.48203500	
C	3.11822800	1.78040500	0.63305400	
C	1.71196100	0.79201200	-1.55791900	
C	1.16058000	1.78456700	-0.76463700	
C	1.86348600	2.30248500	0.32261600	
H	1.15367600	0.41898600	-2.41344000	
O	1.33611500	3.28713000	1.09589300	
H	0.67868600	3.82277900	0.59969200	
C	5.49610800	0.47634000	1.62417500	
H	4.67243000	0.15507100	2.27724900	
H	5.63559100	1.55144700	1.78760000	
C	6.76591700	-0.26963100	2.03808800	
H	7.63169700	0.12056900	1.48958000	
H	6.96379800	-0.07384600	3.09765200	
C	6.62790100	-1.77397800	1.80930800	
H	5.81456900	-2.15474900	2.44451300	
H	7.54319600	-2.29563200	2.11800400	
C	6.31340200	-2.13177500	0.34625500	
C	5.94504700	-3.62092500	0.26997400	
H	5.77509500	-3.94471600	-0.76346800	
H	6.75703000	-4.23293600	0.67896700	
H	5.03584300	-3.83400500	0.84347600	
C	7.56589100	-1.91660100	-0.52077500	
H	8.30563400	-2.68691100	-0.27410900	
H	7.35296100	-2.00391600	-1.59122800	
H	8.04192900	-0.94902800	-0.35428900	
C	6.04862400	0.98235000	-0.78641600	
H	5.88957100	0.70082000	-1.83299500	
H	5.86505700	2.05864400	-0.70075800	
H	7.09740100	0.80639300	-0.54242000	
H	5.34523800	-2.06560400	-2.19235900	

H 4.25228800 -1.68658300 0.55406000<br>H 3.47564000 -0.68197300 -3.16306900<br>O 3.30339300 -2.16414000 -1.65650100<br>O -1.01958600 4.54697600 0.67009100<br>O -1.50868400 3.58006100 1.50345500<br>H -0.73739700 3.01120000 1.70547800	
**Name**	**8-C3-H-OOH (gas phase)**
Cartesian Coordinates	Frequency and Energy
C -1.38317900 1.72132500 0.25538900<br>C -0.17460700 2.38758500 -0.30193300<br>C 0.96061100 1.78260400 -0.69535100<br>C 1.44265600 0.34812600 -0.67229700<br>C 0.93393800 -0.50226300 0.51725100<br>C -0.54316000 -0.67619200 0.48603500<br>C -1.51998200 0.24330400 0.36092000<br>O -2.34246100 2.40629400 0.59566100<br>C -0.35059500 3.87646000 -0.46259700<br>C 1.49541600 0.01624700 1.83167900<br>C 3.00427400 0.05476700 1.82692200<br>C 0.76176300 0.40694200 2.87421000<br>C 3.51408300 0.88727000 0.64336100<br>C 2.98894300 0.34407000 -0.70121300<br>C 3.62735300 -0.98111300 -1.09576500<br>C 2.95773000 -2.04113100 -1.55750000<br>C 5.13357400 -1.01844400 -0.99795900<br>C -2.88017400 -0.39030900 0.30834800<br>C -1.17073700 -2.03279300 0.55183300<br>C -2.68164200 -1.86445600 0.64288700<br>O -0.56470100 -3.07987200 0.49111300<br>H -0.32343200 0.38151100 2.86520200<br>H 1.23999100 0.76010000 3.78305700<br>H 1.35400300 -1.50881700 0.39297000<br>H 1.08349400 -0.12337900 -1.60041400<br>H 1.69201900 2.45982900 -1.14130500<br>H 3.30369600 1.05480600 -1.48173200<br>H 4.60685500 0.92575800 0.64337100<br>H 3.16384800 1.92001500 0.76477400<br>H 3.37502700 0.46406600 2.77105400<br>H 3.38724700 -0.97252700 1.73675000<br>H 3.48985100 -2.93419400 -1.87107100<br>H 1.87600400 -2.08086700 -1.63505200<br>H 5.46309700 -1.06396200 0.04612500<br>H 5.53557900 -1.89216100 -1.51428600<br>H 5.58084400 -0.11940900 -1.43797800<br>H 0.54386900 4.32628700 -0.89702600<br>H -1.20998800 4.09261800 -1.10359400<br>H -0.55307600 4.34527900 0.50417600<br>H -3.13788200 -0.31525800 -0.91116000<br>O -3.90727200 0.20037300 1.00613200<br>H -3.78373800 1.16497500 0.95899100<br>H -2.95047900 -1.97759900 1.70322600<br>C -3.45594700 -2.86725700 -0.20399500<br>H -3.19792600 -3.88553000 0.09476100<br>H -4.53091900 -2.71885300 -0.07740100	Zero-point correction= 0.413527 (Hartree/Particle)<br>Thermal correction to Energy= 0.438446<br>Thermal correction to Enthalpy= 0.439390<br>Thermal correction to Gibbs Free Energy= 0.358540<br>Sum of electronic and zero-point Energies= -1152.243295<br>Sum of electronic and thermal Energies= -1152.218376<br>Sum of electronic and thermal Enthalpies= -1152.217432<br>Sum of electronic and thermal Free Energies= -1152.298281

H -3.21281900 -2.74449100 -1.26332000	
O -2.15449200 0.80225500 -2.41396700	
H -1.43968600 0.24086200 -2.75475300	
O -3.19574600 -0.07499000 -2.20852800	
**Name**	**8-C3-H-OOH (water)**
Cartesian Coordinates	Frequency and Energy
C -1.38770700 1.85386900 -0.05985000	Zero-point correction= 0.412210 (Hartree/Particle)
C -0.09006000 2.56431900 -0.01139900	Thermal correction to Energy= 0.437042
C 1.10572400 1.98120800 -0.21236500	Thermal correction to Enthalpy= 0.437986
C 1.45027900 0.54476900 -0.52715600	Thermal correction to Gibbs Free Energy= 0.357664
C 0.88535900 -0.46658000 0.49668300	Sum of electronic and zero-point Energies= -1152.270681
C -0.60007000 -0.52465400 0.51310500	Sum of electronic and thermal Energies= -1152.245849
C -1.54514700 0.40273800 0.22952200	Sum of electronic and thermal Enthalpies= -1152.244905
O -2.41662700 2.49626200 -0.28913200	Sum of electronic and thermal Free Energies= -1152.325227
C -0.21025200 4.05674000 0.16515400	
C 1.52752600 -0.28832900 1.86136000	
C 3.02843400 -0.41627400 1.80013400	
C 0.85085400 -0.01719800 2.98004500	
C 3.60254400 0.57588500 0.77978400	
C 2.98331300 0.39149900 -0.62101800	
C 3.45754100 -0.87959300 -1.31345500	
C 2.66131400 -1.72081200 -1.98427200	
C 4.94485200 -1.12428400 -1.25957300	
C -2.90502000 -0.22137800 0.16717200	
C -1.28878000 -1.81755000 0.80265900	
C -2.78455500 -1.59358300 0.81113800	
O -0.72243300 -2.88385800 0.97596400	
H -0.23117300 0.08989400 2.99054700	
H 1.37280100 0.10027500 3.92627200	
H 1.20602900 -1.46136100 0.15629000	
H 1.00163000 0.31220700 -1.50243000	
H 1.95467700 2.66607200 -0.21511100	
H 3.35650400 1.21720000 -1.24561400	
H 4.69053800 0.48510200 0.73288100	
H 3.38969200 1.59461300 1.12573500	
H 3.46315200 -0.24383700 2.78886700	
H 3.28677500 -1.44009400 1.49190000	
H 3.08317900 -2.57880200 -2.50151600	
H 1.58366400 -1.59964500 -2.05126100	
H 5.25783400 -1.42914400 -0.25454200	
H 5.23477200 -1.91182600 -1.95877700	
H 5.50112900 -0.21236500 -1.50543500	
H 0.78007900 4.50590200 0.25772300	
H -0.72136800 4.51025700 -0.68933100	
H -0.79384200 4.29905200 1.05862300	
H -3.00420200 -0.41478700 -1.06274300	
O -4.01348500 0.49428300 0.59538800	
H -3.91029900 1.40870800 0.27515200	
H -3.07135100 -1.46743300 1.86611100	
C -3.58642200 -2.72246600 0.17898300	
H -3.44290100 -3.64804000 0.74120500	
H -4.65111300 -2.47618600 0.18596600	
H -3.27179700 -2.88862700 -0.85501600	
O -1.70832400 0.15173000 -2.65602000	

H	-0.98205200	-0.49032600	-2.54995300	
O	-2.84922100	-0.55801300	-2.37438300	
**Name**				**8-C3-H-OOH (pentyl ethanoate)**
Cartesian Coordinates				Frequency and Energy
C	-1.38635800	1.75397100	0.19705500	Zero-point correction= 0.412842 (Hartree/Particle)
C	-0.14079300	2.43974600	-0.22647200	Thermal correction to Energy= 0.437763
C	1.01054500	1.84425100	-0.59180500	Thermal correction to Enthalpy= 0.438707
C	1.45469900	0.40118200	-0.65575700	Thermal correction to Gibbs Free Energy= 0.357743
C	0.93650500	-0.49217100	0.49685900	Sum of electronic and zero-point Energies= -1152.272386
C	-0.54295900	-0.64425000	0.48015900	Sum of electronic and thermal Energies= -1152.247465
C	-1.51801900	0.27577600	0.32461500	Sum of electronic and thermal Enthalpies= -1152.246521
O	-2.38650400	2.42919200	0.43940000	Sum of electronic and thermal Free Energies= -1152.327485
C	-0.27833800	3.94033600	-0.29007300	
C	1.51971600	-0.05311300	1.83096400	
C	3.02832200	-0.05603400	1.81586700	
C	0.79970400	0.31450100	2.89280200	
C	3.55329900	0.81330100	0.66581200	
C	3.00021300	0.35621900	-0.69934200	
C	3.59606900	-0.96421500	-1.17050600	
C	2.89182400	-1.96894700	-1.70286200	
C	5.09783700	-1.06404800	-1.06463100	
C	-2.87847800	-0.36038900	0.29179200	
C	-1.17912700	-1.99143600	0.62137900	
C	-2.68665400	-1.81611700	0.70010600	
O	-0.57809100	-3.04553900	0.63265900	
H	-0.28683000	0.32450300	2.88632900	
H	1.29157900	0.61349500	3.81490300	
H	1.33965500	-1.49796000	0.32111900	
H	1.06681800	-0.00728600	-1.60077600	
H	1.77710000	2.53906700	-0.93900000	
H	3.32895800	1.09784800	-1.44358300	
H	4.64657500	0.81486600	0.65858000	
H	3.24021300	1.85080900	0.83892300	
H	3.41614800	0.30230200	2.77385500	
H	3.38092100	-1.08884000	1.67772300	
H	3.39493000	-2.86013100	-2.06890700	
H	1.80969800	-1.95907600	-1.79548100	
H	5.41539200	-1.17707700	-0.02173800	
H	5.47115400	-1.92439400	-1.62474600	
H	5.58195000	-0.15958000	-1.45166500	
H	0.65801700	4.39601900	-0.61766100	
H	-1.07506500	4.23053400	-0.98152200	
H	-0.54388400	4.34764700	0.69016400	
H	-3.13251900	-0.34414900	-0.92967000	
O	-3.91541600	0.26389500	0.94648700	
H	-3.78136200	1.22418400	0.84715300	
H	-2.95418200	-1.87576400	1.76525500	
C	-3.46545000	-2.85812000	-0.09255500	
H	-3.26644300	-3.85771100	0.30086900	
H	-4.53907100	-2.66390700	-0.02229300	
H	-3.17883900	-2.83951000	-1.14819200	
O	-2.18063200	0.66323000	-2.53589700	
H	-1.48883800	0.05104900	-2.84175800	
O	-3.24528300	-0.15572800	-2.23618600	

**Name**	**9-C13-H-OOH (gas phase)**
Cartesian Coordinates	Frequency and Energy
C 2.23878300 0.74773100 1.26299200	Zero-point correction= 0.461677 (Hartree/Particle)
C 1.12491900 1.19798600 0.36496800	Thermal correction to Energy= 0.489119
C 1.40334100 0.62626300 -1.01571700	Thermal correction to Enthalpy= 0.490063
C 2.83902400 0.12078300 -0.87044000	Thermal correction to Gibbs Free Energy= 0.406422
C 3.30000300 0.16714500 0.38511100	Sum of electronic and zero-point Energies= -1229.804053
C 4.61097200 -0.24976100 0.95858800	Sum of electronic and thermal Energies= -1229.776612
O 2.30113400 0.86979100 2.46602400	Sum of electronic and thermal Enthalpies= -1229.775668
C 0.27156700 2.13814100 0.78897700	Sum of electronic and thermal Free Energies= -1229.859308
C -0.79530300 2.89738600 0.00232400	
C -1.53254800 3.85201900 0.93592600	
C -1.76980700 1.92210900 -0.67238300	
C -2.60532100 1.09709200 0.31595200	
C -3.08833100 -0.19496200 -0.30128200	
C 0.55290600 -0.60262000 -1.44514500	
C 0.25035700 -1.75886000 -0.53004800	
C -0.90698200 -1.54942300 0.44553900	
C -2.25436300 -1.46220800 -0.26437500	
C -3.54791700 -1.37460500 0.51565800	
C -3.50238600 -1.32012400 2.03018700	
C -4.72407700 -2.16953000 -0.01440200	
C 0.24405400 -3.08411500 -1.26272100	
O 0.21108700 -0.64181400 -2.61341500	
H 0.41596800 2.43179800 1.83129800	
H -3.63416600 -0.04392900 -1.23288700	
H 1.24757300 -1.93833800 0.32223700	
H 3.37427600 -0.22891300 -1.75014800	
H -2.42010700 2.50995700 -1.33071100	
H -1.20239500 1.24841400 -1.32598200	
H -3.46748600 1.68278300 0.65754000	
H -2.01177900 0.89248700 1.21422800	
H -0.90290000 -2.40906300 1.12816200	
H -0.71889000 -0.66119100 1.05484800	
H 5.09781200 0.60319100 1.44036100	
H 4.44627400 -0.99442900 1.74637900	
H 5.28575900 -0.64910700 0.19640900	
H -2.30492900 4.38564900 0.37548400	
H -1.99977300 3.31901600 1.76874800	
H -0.82731200 4.58477300 1.33692000	
H -3.48572700 -2.33539900 2.44249000	
H -2.62788900 -0.79407100 2.41869400	
H -4.39497400 -0.81797200 2.41956500	
H -4.68831200 -3.20502500 0.34276100	
H -5.67082000 -1.72949800 0.31918900	
H -4.72794800 -2.18831100 -1.10858200	
H 0.04364400 -3.89526100 -0.55830100	
H -0.51975200 -3.08972300 -2.04720000	
H 1.20926700 -3.27241000 -1.74096400	
O 1.31409600 1.59234900 -2.03659100	
H 0.93432400 1.14253100 -2.81347500	
O -0.17185300 3.72868700 -0.96484800	
H 0.40170400 3.17263200 -1.51602400	
H -2.31359100 -2.07639700 -1.16054600	

O 2.94814600 -3.05311100 0.27002100	
H 3.63266100 -2.38087500 0.11089600	
O 2.04440800 -2.40415300 1.08885300	
**Name**	**9-C13-H-OOH (water)**
Cartesian Coordinates	Frequency and Energy
C -2.26475600 0.72641000 -1.24486400	Zero-point correction= 0.460137 (Hartree/Particle)
C -1.15024500 1.19475300 -0.36785600	Thermal correction to Energy= 0.487642
C -1.41895800 0.62961400 1.01912200	Thermal correction to Enthalpy= 0.488587
C -2.84242800 0.09783300 0.88265700	Thermal correction to Gibbs Free Energy= 0.405035
C -3.31635400 0.15114600 -0.36997000	Sum of electronic and zero-point Energies= -1229.837311
C -4.63028200 -0.28302900 -0.91924400	Sum of electronic and thermal Energies= -1229.809805
O -2.32152700 0.82030400 -2.46307500	Sum of electronic and thermal Enthalpies= -1229.808861
C -0.29508000 2.12633800 -0.80858100	Sum of electronic and thermal Free Energies= -1229.892412
C 0.77292300 2.89455900 -0.03466500	
C 1.48232900 3.86174700 -0.97211900	
C 1.76444900 1.93981900 0.63681300	
C 2.59307000 1.11176100 -0.35295700	
C 3.09876800 -0.16036000 0.28756700	
C -0.53549900 -0.57088400 1.45285500	
C -0.23619400 -1.74635800 0.56032000	
C 0.92668400 -1.55389400 -0.41356100	
C 2.27441000 -1.43539500 0.29046000	
C 3.55781100 -1.35497100 -0.50800300	
C 3.49208100 -1.33751700 -2.02203800	
C 4.74554500 -2.12844000 0.02602000	
C -0.23104500 -3.05554000 1.31945000	
O -0.14731900 -0.57283600 2.61025300	
H -0.42749200 2.41266800 -1.85370200	
H 3.65780800 0.01540700 1.20700700	
H -1.21530600 -1.93050000 -0.28496400	
H -3.36331500 -0.28332300 1.75702700	
H 2.42362200 2.53954300 1.27569300	
H 1.20775200 1.27094700 1.30394900	
H 3.44185000 1.70593900 -0.71249900	
H 1.99016200 0.88373800 -1.23853700	
H 0.93173800 -2.43691900 -1.06528500	
H 0.73079400 -0.68763700 -1.05142300	
H -5.14847800 0.56479500 -1.37813900	
H -4.47796300 -1.03101200 -1.70602000	
H -5.26499700 -0.70598800 -0.13762400	
H 2.26029400 4.40367200 -0.42635700	
H 1.94458400 3.32750900 -1.80640400	
H 0.76500700 4.58126700 -1.37824200	
H 3.45996400 -2.36332100 -2.40775700	
H 2.61829400 -0.81003400 -2.41114600	
H 4.38647500 -0.85645700 -2.43476200	
H 4.71344200 -3.17206400 -0.30873800	
H 5.68552600 -1.69023600 -0.33030400	
H 4.76056400 -2.12171100 1.12040400	
H -0.11011000 -3.88586900 0.61837100	
H 0.59732700 -3.08639900 2.03610700	
H -1.16012400 -3.19220600 1.88003900	
O -1.35402600 1.61885100 2.02542500	

H	-0.95852100	1.20583400	2.81579000	
O	0.13307400	3.70813300	0.95709600	
H	-0.38519000	3.11310800	1.53012500	
H	2.34983700	-2.03248500	1.19713700	
O	-2.87969200	-3.15478400	-0.34457200	
H	-3.56946600	-2.52370300	-0.06632500	
O	-2.00144000	-2.40218400	-1.09910800	
**Name**				**9-C3-H-OOH (pentyl ethanoate)**
Cartesian Coordinates				Frequency and Energy
C	-2.22125100	0.75695500	-1.28832100	Zero-point correction= 0.460995 (Hartree/Particle)
C	-1.11759500	1.20332200	-0.37682200	Thermal correction to Energy= 0.488268
C	-1.41590800	0.62740200	0.99815100	Thermal correction to Enthalpy= 0.489212
C	-2.84658500	0.11853600	0.83189400	Thermal correction to Gibbs Free Energy= 0.406487
C	-3.29428400	0.17706600	-0.42919200	Sum of electronic and zero-point Energies= -1229.834904
C	-4.60458100	-0.23548200	-1.00552400	Sum of electronic and thermal Energies= -1229.807631
O	-2.26145100	0.87939600	-2.49566600	Sum of electronic and thermal Enthalpies= -1229.806687
C	-0.25457700	2.14155900	-0.78637700	Sum of electronic and thermal Free Energies= -1229.889412
C	0.80665700	2.89188000	0.01582300	
C	1.55055500	3.85438900	-0.90188400	
C	1.77255000	1.90862900	0.68928500	
C	2.61401800	1.09513000	-0.30281000	
C	3.10126700	-0.20066500	0.30335600	
C	-0.57367200	-0.59864300	1.45539700	
C	-0.24416500	-1.76108500	0.55695100	
C	0.90699900	-1.54741300	-0.42643600	
C	2.26327700	-1.46608400	0.26872000	
C	3.54523200	-1.37515700	-0.53088800	
C	3.47650600	-1.30802800	-2.04390600	
C	4.72654400	-2.17779700	-0.02530400	
C	-0.21440100	-3.07681300	1.30498100	
O	-0.27209300	-0.63096500	2.63586300	
H	-0.37646700	2.44232300	-1.82920200	
H	3.66294400	-0.05817800	1.22695000	
H	-1.23916100	-1.96195800	-0.28224400	
H	-3.39265500	-0.24640400	1.69921300	
H	2.42093500	2.48462000	1.36020800	
H	1.19538800	1.22821900	1.32752700	
H	3.47502100	1.68940900	-0.63260400	
H	2.02614100	0.89643800	-1.20554900	
H	0.89963900	-2.40717400	-1.10901700	
H	0.71689900	-0.65828400	-1.03335700	
H	-5.11413700	0.62974800	-1.44183500	
H	-4.44943800	-0.94976400	-1.82246500	
H	-5.26036000	-0.67491300	-0.24877000	
H	2.32561200	4.37981300	-0.33598800	
H	2.01969700	3.32732200	-1.73736100	
H	0.85148400	4.59289500	-1.30545300	
H	3.45474100	-2.32049300	-2.46435900	
H	2.59592800	-0.77907000	-2.41601300	
H	4.36378500	-0.80297000	-2.44359600	
H	4.68204000	-3.21142500	-0.38889500	
H	5.67055400	-1.74008000	-0.37183300	
H	4.74829300	-2.20394400	1.06894100	
H	-0.01140800	-3.89447600	0.60813500	

H	0.56380100	-3.07124300	2.07571800	
H	-1.16827500	-3.26993600	1.80449900	
O	-1.34867900	1.60215300	2.01631900	
H	-1.01099000	1.15200700	2.81270400	
O	0.16995300	3.71197000	0.99222000	
H	-0.39563700	3.13612600	1.53288800	
H	2.33733600	-2.09153600	1.15586700	
O	-2.97567100	-3.05322200	-0.25598900	
H	-3.64171200	-2.35860600	-0.10327500	
O	-2.03824800	-2.43741600	-1.06067400	

Table S5. The method to calculate rate constant following the conventional transition state theory

The rate constant (k) was calculated by using the conventional transition state theory (TST) and 1M standard state as:[1-6]

$$k = \sigma\kappa \frac{k_B T}{h} e^{-(\Delta G^{\neq})/RT}$$

Where k_B and h are the Boltzmann and Planck constants, respectively, $\Delta G^{\neq}$ is Gibbs free energy of activation of the studied reaction, σ is the reaction symmetry number that represents reaction path degeneracy (which was calculated following the literature[7,8]), the number of possible different but equivalent reaction pathways, and κ accounts for tunneling corrections which were calculated using Eckart barrier[9]. The Marcus Theory was used to estimate the reaction barriers of SET reactions[10-13]. To avoid over-penalizing entropy losses in solution, in this study the solvent cage effects were included following the corrections proposed by Okuno[14], adjusted with the free volume theory according to the Benson correction[15,16]. These corrections have been successfully used to study the radical scavenging activity of antioxidants in solution[16-19] and are in good agreement with activity data independently obtained by Ardura *et al*[20]. For rate constants that were close to the diffusion limit a correction was applied to yield realistic results[16]. The apparent rate constants (k_{app}) were calculated following the Collins–Kimball theory in the solvents at 298.15K[21]; the steady-state Smoluchowski rate constant (k_D) for an irreversible bimolecular diffusion–controlled reaction was calculated following the literature[16,22].

For the species that have multiple conformers, all of these were investigated and the conformer with the lowest electronic energy was included in the analysis. The hindered internal rotation treatment was also applied to the single bonds to ensure that the obtained conformer has the lowest electronic energy[23,24]. All transition states were characterized by the existence of only one single imaginary frequency. Intrinsic coordinate calculations (IRCs) were performed to ensure that each transition state is corrected.

References

1. M. G. Evans and M. Polanyi, *Trans. Faraday Soc.*, 1935, **31**, 875-894.
2. H. Eyring, *J. Chem. Phys.*, 1935, **3**, 107-115.
3. D. G. Truhlar, W. L. Hase and J. T. Hynes, *J. Phys. Chem.*, 1983, **87**, 2664-2682.
4. Y. Zhao, N. E. Schultz and D. G. Truhlar, *J. Chem. Theory Comput.*, 2006, **2**, 364-382.
5. T. Furuncuoglu, I. Ugur, I. Degirmenci and V. Aviyente, *Macromolecules*, 2010, **43**, 1823-1835.
6. E. Vélez, J. Quijano, R. Notario, E. Pabón, J. Murillo, J. Leal, E. Zapata and G. Alarcón, *J. Phys. Org. Chem.*, 2009, **22**, 971-977.
7. E. Pollak and P. Pechukas, *J. Am. Chem. Soc.*, 1978, **100**, 2984-2991.
8. A. Fernández-Ramos, B. A. Ellingson, R. Meana-Pañeda, J. M. Marques and D. G. Truhlar, *Theor. Chem. Acc.*, 2007, **118**, 813-826.
9. C. Eckart, *Phy. Rev.*, 1930, **35**, 1303.
10. R. A. Marcus, *Annu. Rev. Phys. Chem.*, 1964, **15**, 155-196.
11. R. A. Marcus, *Rev. Mod. Phys.*, 1993, **65**, 599.
12. Y. Lu, A. Wang, P. Shi and H. Zhang, *PloS one*, 2017, **12**, e0169773.
13. Y. Lu, A. Wang, P. Shi, H. Zhang and Z. Li, *PloS one*, 2015, **10**, e0133259.
14. Y. Okuno, *Chem.: Eur. J.*, 1997, **3**, 212-218.
15. S. Benson, *The foundations of chemical kinetics:* , Malabar, Florida, 1982.
16. A. Galano and J. R. Alvarez-Idaboy, *J. Comput. Chem.*, 2013, **34**, 2430-2445.
17. C. Iuga, J. R. Alvarez-Idaboy and A. Vivier-Bunge, *J. Phys. Chem. B*, 2011, **115**, 12234-12246.
18. J. R. Alvarez-Idaboy, L. Reyes and N. Mora-Diez, *Org. Biomol. Chem.*, 2007, **5**, 3682-3689.
19. J. R. Alvarez-Idaboy, L. Reyes and J. Cruz, *Org. Lett.*, 2006, **8**, 1763-1765.
20. D. Ardura, R. López and T. L. Sordo, *J. Phys. Chem. B*, 2005, **109**, 23618-23623.
21. F. C. Collins and G. E. Kimball, *J. Colloid Sci.* , 1949, **4**, 425-437.
22. M. Von Smoluchowski, *Z. Phys. Chem*, 1917, **92**, 129-168.
23. T. V.-T. Mai, M. v. Duong, X. T. Le, L. K. Huynh and A. Ratkiewicz, *Struct. Chem.*, 2014, **25**, 1495-1503.
24. T. H. Le, T. T. Tran and L. K. Huynh, *Chemom. Intell. Lab. Syst.*, 2018, **172**, 10-16.
